# Super‐Resolution MRI–Derived Tumor‐Stroma Ratio Phenotyping for Prognostic and Therapeutic Stratification in Pancreatic Ductal Adenocarcinoma

**DOI:** 10.1002/mco2.71039

**Published:** 2026-10-09

**Authors:** Xingxing Wang, Zongyu Xie, Hanxiao Li, Qiying Tang, Jianjun Zhou, Jingcheng Huang, Shiwu Fang, Dalong Li, Lei Li, Mengsu Zeng, Minping Hong, Qiuyang Zhu, Yaolin Xu, Haitao Sun

**Affiliations:** ^1^ Department of Pathology Zhongshan Hospital Fudan University Shanghai China; ^2^ Department of Radiology The First Affiliated Hospital of Bengbu Medical University Bengbu China; ^3^ Department of Radiology Zhongshan Hospital, Fudan University Shanghai Institute of Medical Imaging Shanghai China; ^4^ Department of Radiology Zhongshan Hospital (Xiamen), Fudan University Xiamen China; ^5^ Department of Radiology Second Affiliated Hospital of Bengbu Medical University Bengbu China; ^6^ Department of Radiology Fengyang County People's Hospital Chuzhou China; ^7^ Department of Radiology Jiaxing Hospital of Traditional Chinese Medicine Affiliated to Zhejiang Chinese Medical University Jiaxing China; ^8^ School of Instrument and Electronics North University of China Shanxi China; ^9^ Ceyear Technologies Co. Ltd. Shandong China; ^10^ Department of Pancreatic Surgery Zhongshan Hospital Fudan University Shanghai China; ^11^ Cancer Center Zhongshan Hospital, Fudan University Shanghai China; ^12^ Anhui Key Laboratory of Digital Medicine and Intelligent Health Bengbu China

**Keywords:** pancreatic ductal adenocarcinoma, tumor‐stroma ratio, radiomics, super‐resolution MRI, tumor microenvironment

## Abstract

Pancreatic ductal adenocarcinoma (PDAC) exhibits profound stromal heterogeneity that influences tumor progression, recurrence risk, and therapeutic response; however, stromal phenotypes are currently assessed by postoperative histopathology. Here, we developed a super‐resolution MRI (srMRI)–based radiomics model to enable noninvasive tumor‐stroma ratio (TSR) phenotyping in PDAC. Using multicenter cohorts comprising 737 patients, we constructed a srMRI‐derived TSR‐radscore capable of predicting pathological TSR with high accuracy (AUC = 0.870 and 0.791 in training and external validation cohorts). The TSR‐radscore independently stratified recurrence‐free survival and, when integrated with clinicopathological variables, improved early recurrence prediction in an independent surgical cohort. In a locally advanced PDAC cohort, srMRI‐derived TSR was associated with distinct therapeutic outcomes, with H‐TSR tumors showing improved survival under systemic therapy and an association with outcomes following immune checkpoint inhibitor‐based combination therapy. Transcriptomic analyses revealed that L‐TSR tumors were enriched in extracellular matrix remodeling and TGF‐β–related signaling pathways. Single‐cell profiling further uncovered distinct cancer‐associated fibroblast and T‐cell states across TSR phenotypes, linking imaging‐derived TSR patterns to underlying stromal and immune microenvironmental programs. Collectively, srMRI‐derived TSR phenotyping provides a noninvasive and biologically interpretable biomarker for stromal characterization in PDAC, enabling improved recurrence‐risk stratification and pre‐treatment clinical assessment.

## Graphical abstract

Super‐resolution MRI–derived radiomics enables noninvasive tumor‐stroma ratio (TSR) phenotyping in pancreatic ductal adenocarcinoma. The imaging‐derived TSR‐radscore predicts early recurrence risk and stratifies therapeutic outcomes in locally advanced disease. Multi‐omics analyses further link TSR phenotypes to extracellular matrix remodeling, stromal activation, and distinct immune microenvironmental programs.

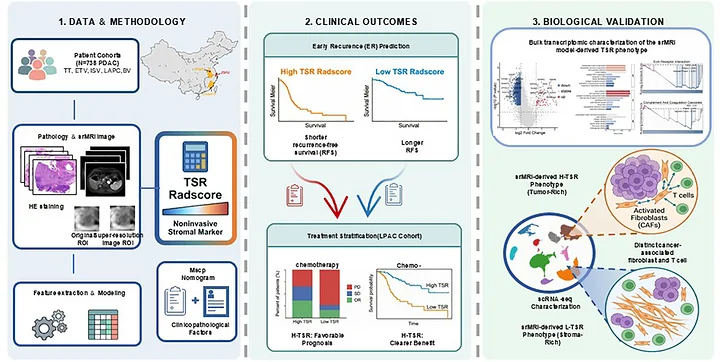

## 1 Introduction

Pancreatic ductal adenocarcinoma (PDAC) is one of the deadliest solid malignancies, characterized by rapid progression, early dissemination, and dismal prognosis [1, 2]. Even among patients who undergo curative‐intent resection, nearly half experience early recurrence (ER, <1 year), and long‐term survival remains rare [3, 4]. Therapeutic progress in advanced or recurrent PDAC remains disappointing. Targeted agents and immunotherapies have shown minimal clinical efficacy, and the survival benefit of chemotherapy or chemoimmunotherapy remains highly heterogeneous across patients. Thus, accurate pretreatment risk stratification is critically needed to identify high‐risk patients early and optimize therapeutic decision‐making in PDAC [5, 6].

Stromal heterogeneity is a hallmark of PDAC and contributes substantially to tumor progression, recurrence, and therapeutic response. One of the most widely used pathological measures of stromal heterogeneity is the tumor‐stroma ratio (TSR), which has shown prognostic value across multiple malignancies, including PDAC [7, 8]. Multiple PDAC cohorts have linked high TSR to increased lymph node metastasis and poor survival [9, 10]. Notably, the PDAC stroma exerts a context‐dependent, dual role in tumor progression. Jiang et al. have reported that depletion of ECM/collagen accelerated tumor growth and reduced survival, highlighting a restraining role of stromal components in PDAC progression [11]. Emerging evidence further links distinct CAF states and extracellular matrix (ECM) remodeling to differences in tumor progression and therapeutic response. Beyond prognosis, accumulating evidence suggests that stromal composition also modulates therapeutic vulnerability [12]. Dense or remodeled stroma affects drug delivery, immune infiltration, and radiosensitivity, suggesting that TSR may modulate systemic therapy response and holds potential therapeutic relevance [13].

Currently, TSR assessment depends on postoperative histopathology, which is invasive, subject to sampling bias, and unsuitable for preoperative or longitudinal monitoring [14]. These limitations highlight the urgent need for noninvasive and reproducible methods capable of assessing TSR status and capturing stromal heterogeneity before treatment [15, 16]. Radiomics provides a noninvasive strategy to capture imaging features reflective of stromal architecture. To date, only a few radiomic studies have attempted to predict TSR in PDAC, with most relying on CT, whereas MRI, despite its superior capacity to depict the tumor microenvironment, has received little attention [10, 17, 18]. Few studies have correlated radiomic signatures directly to TSR or evaluated their relevance for ER risk or chemotherapy benefit in large PDAC cohorts. Moreover, existing models provide limited biological interpretability, as the biological underpinnings of imaging‐derived TSR surrogates remain unclear. Integrating imaging with transcriptomic and single‐cell analyses may improve the biological interpretability of stromal phenotypes.

In this study, we developed a TSR‐guided super‐resolution MRI radiomics signature to noninvasively characterize stromal phenotypes in PDAC. We then evaluated its ability to predict ER and chemotherapy response across multicenter cohorts. To explore the biological basis of imaging‐derived TSR phenotypes, we integrated bulk transcriptomic analysis with single‐cell data, linking imaging features to underlying stromal and immune microenvironmental states (Figure 1).

**FIGURE 1:**
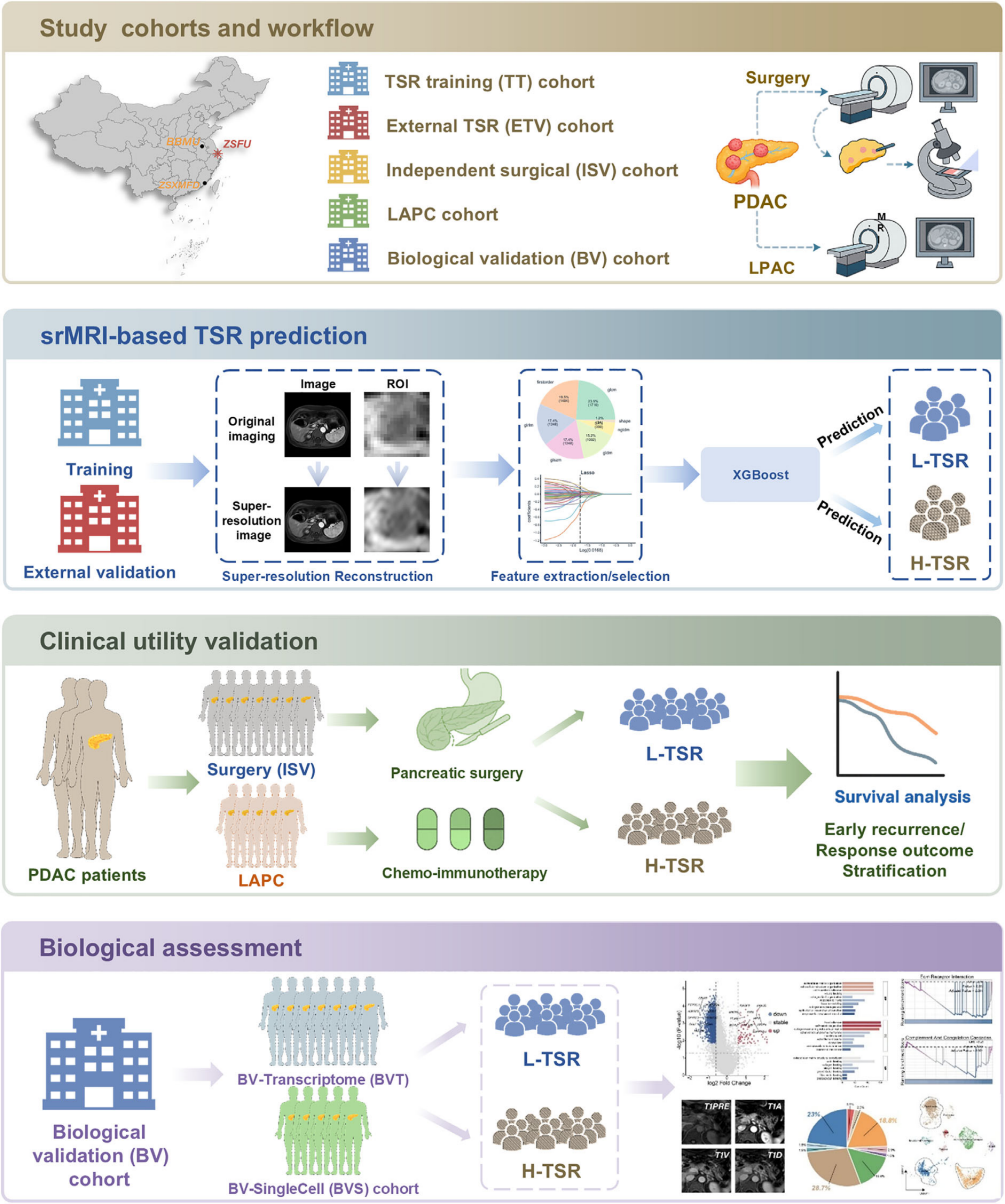
Study workflow integrating data cohorts, srMRI‐based tumor stroma ratio (TSR) prediction, clinical utility assessment, and biological assessment. The base map of China was generated based on a standard map (approval number GS(2024)0650).

## 2 Results

### 2.1 Baseline Characteristics

A total of 737 patients with pathologically confirmed PDAC were included in this study. Among them, 657 patients were analyzed in the clinical and imaging cohorts, including 166 in the TT cohort, 92 in the ETV cohort, 317 in the independent surgery validation (ISV) cohort, and 82 in the LAPC cohort, whereas 80 treatment‐naïve patients were included in the biological validation cohort for bulk (BVT cohort, *n* = 68) and single‐cell transcriptomic (BVS cohort, *n* = 12) analyses (Table 1 and Figure 2). The interobserver agreement for pathological TSR assessment was excellent (Cohen's *κ* = 0.899, *p* < 0.001). Most baseline clinicopathological variables did not differ significantly across cohorts, although variations were observed in the distributions of pT and pN stages (*p* < 0.05). In the pathological TSR stratification, high‐TSR (>1) cases accounted for 65.1% (108/166) in TT and 63.0% (58/92) in ETV, with no significant differences in other clinicopathological variables between these cohorts (all *p* > 0.05) (Table S2). The LAPC cohort (*n* = 82) had a median age of 68 years, with 79.3% of tumors located in the pancreatic head/uncinate. Overall, 45.1% of patients received immunotherapy and 67.1% experienced disease progression (Table S3).

**TABLE 1:** Baseline demographic and clinicopathological characteristics.

Parameters	TT (*n* = 166)	ETV (*n* = 92)	ISV (*n* = 317)
Age (years)	65.5 [60.0, 70.0]	65.5 [57.5, 71.5]	66.0 [59.0, 72.0]
Sex			
Female	71 (42.8%)	35 (38.0%)	139 (43.8%)
Male	95 (57.2%)	57 (62.0%)	178 (56.2%)
DM			
Absence	112 (67.5%)	61 (66.3%)	232 (73.2%)
Presence	54 (32.5%)	31 (33.7%)	85 (26.8%)
HBP			
Absence	91 (54.8%)	59 (64.1%)	194 (61.2%)
Presence	75 (45.2%)	33 (35.9%)	123 (38.8%)
BMI (kg/m^2^)	22.7 [20.4, 24.3]	22.9 [20.9, 25.2]	22.6 [20.8, 24.6]
TB (µmol/L)			
≤20.4	111 (66.9%)	61 (66.3%)	197 (62.1%)
>20.4	55 (33.1%)	31 (33.7%)	120 (37.9%)
Albumin (g/L)			
>35	153 (92.2%)	84 (91.3%)	284 (89.6%)
≤35	13 (7.8%)	8 (8.7%)	33 (10.4%)
CA19‐9 (U/mL)			
≤100	78 (47.0%)	34 (37.0%)	117 (36.9%)
>100	88 (53.0%)	58 (63.0%)	200 (63.1%)
Location			
Head/Uncinate	94 (56.6%)	53 (57.6%)	212 (66.9%)
Body/Tail	72 (43.4%)	39 (42.4%)	105 (33.1%)
Tumor size (cm)	2.5 [2.0, 3.2]	2.8 [2.2, 3.7]	2.7 [2.1, 3.4]
pT Stage			
T1	48 (28.9%)	20 (21.7%)	84 (26.5%)
T2	94 (56.6%)	48 (52.2%)	192 (60.6%)
T3	24 (14.5%)	24 (26.1%)	41 (12.9%)
pN Stage			
N0	89 (53.6%)	59 (64.1%)	170 (53.6%)
N1	64 (38.6%)	20 (21.7%)	126 (39.7%)
N2	13 (7.8%)	13 (14.1%)	21 (6.6%)
PD			
Well/Moderately‐differentiated	84 (50.6%)	48 (52.2%)	158 (49.8%)
Poor	82 (49.4%)	44 (47.8%)	159 (50.2%)
PFI			
Absence	36 (21.7%)	21 (22.8%)	69 (21.8%)
Presence	130 (78.3%)	71 (77.2%)	248 (78.2%)
PVI			
Absence	102 (61.4%)	62 (67.4%)	222 (70.0%)
Presence	64 (38.6%)	30 (32.6%)	95 (30.0%)
PNI			
Absence	23 (13.9%)	15 (16.3%)	45 (14.2%)
Presence	143 (86.1%)	77 (83.7%)	272 (85.8%)
AC			
Without AC	26 (15.7%)	14 (15.2%)	92 (29.0%)
With AC	140 (84.3%)	78 (84.8%)	225 (71.0%)
RFS (months)	15.8 [7.5, 31.3]	6.3 [3.4, 10.6]	13.6 [6.1, 26.0]
Recurrence			
No	57 (34.3%)	40 (43.5%)	126 (39.7%)
Yes	109 (65.7%)	52 (56.5%)	191 (60.3%)

*Note*: Continuous variables are presented as median [interquartile range, Q1–Q3], and categorical variables as *n* (%).

Abbreviations: TT = TSR training; ETV = external TSR validation; ISV = independent surgery validation; PDAC = pancreatic ductal adenocarcinoma; DM = diabetes; HBP = high blood pressure; BMI = body mass index; TB = total bilirubin; CA19‐9 = cancer antigen 19‐9; PD = pathological differentiation; PVI = pathological lymphovascular invasion; PFI = peripancreatic fat invasion; PNI = perineural invasion; AC = Adjuvant treatment; RFS = recurrence‐free survival.

**FIGURE 2:**
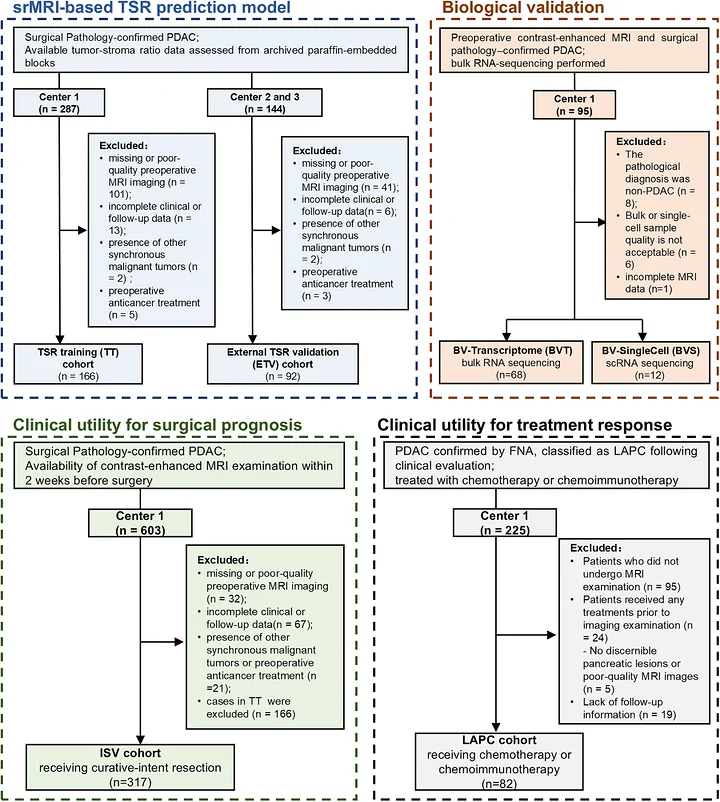
Flowchart of patient enrollment and selection.

### 2.2 TSR Predictive Performance and Interpretability of the srMRI Model

Representative cases demonstrated that SR reconstruction provided a clearer and better‐defined ROI than the original MRI (Figure 3A and Figures S1–S2). A total of 7182 radiomics features were extracted from six MRI sequences, mainly from GLCM (23.9%), first‐order (19.5%), GLRLM and GLSZM (each 17.4%), and GLDM (15.2%) categories. Sequential feature selection (univariate filtering, correlation removal, mRMR, and LASSO) retained 30 predictors for TSR‐radscore construction (Figure 3B,C and Table S4). SHAP analysis further identified the major contributors to the model, with higher values of *log‐sigma‐5‐0‐mm_3D_firstorder_Skewness_T1D* and *wavelet_HHH_firstorder_Skewness_T2* markedly increasing the TSR‐radscore and corresponding to the stroma‐poor phenotype (TSR > 1). In addition, higher values of GLDM and GLCM descriptors (e.g., *LargeDependenceHighGrayLevelEmphasis, SmallAreaEmphasis, Contrast, and Imc1*) increased the TSR‐radscore, whereas lower intensity‐ or uniformity‐related features (e.g., median and strength) were associated with stroma‐rich tumors (TSR ≤ 1). Similar SHAP patterns were observed in the ETV cohort (Figure S3A).

**FIGURE 3:**
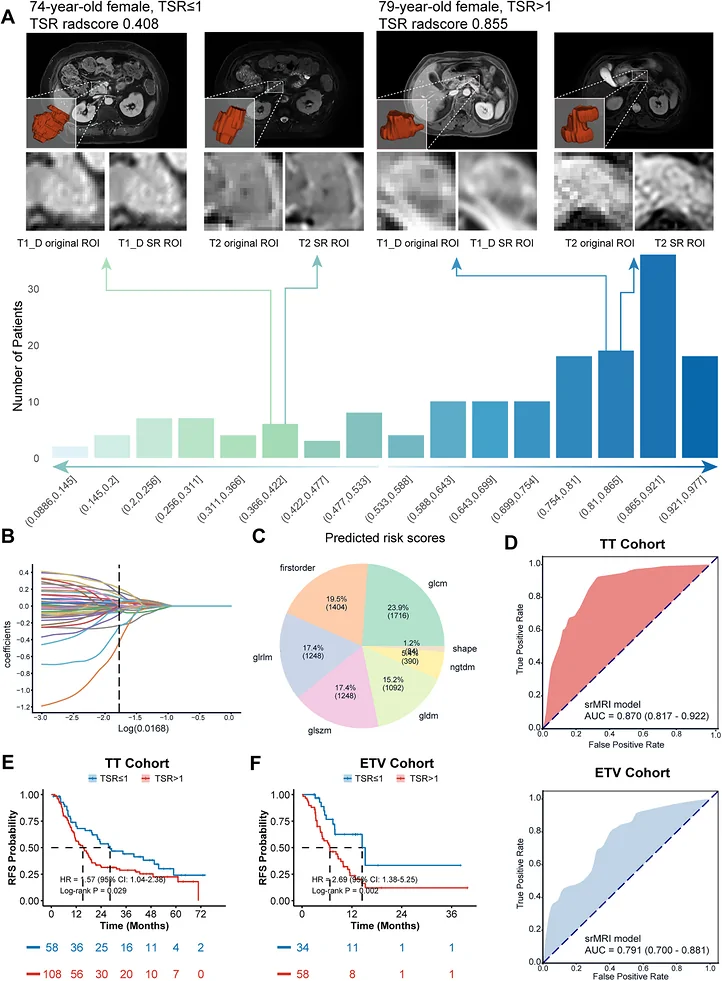
Development and validation of the srMRI‐based TSR model: (A) TSR‐radscores of two representative patients are shown, and the histogram illustrates the overall score distribution across the cohort. (B) LASSO regression identifying the optimal λ for selecting non‐redundant radiomic predictors. (C) Composition of the 7,182 extracted radiomic features across seven categories, including first‐order, GLCM, GLDM, GLRLM, GLSZM, shape, and NGTDM features. (D) ROC curves demonstrating the discriminative performance of the srMRI model in the TT and ETV cohorts, with AUCs of 0.870 and 0.791, respectively. (E,F) Kaplan–Meier curves for RFS stratified by pathology‐confirmed TSR phenotype (TSR ≤ 1 vs. TSR > 1) in the TT (E) and ETV (F) cohorts, showing significantly poorer RFS in the TSR > 1 group.

The developed srMRI model integrating six sequences and built with the XGBoost algorithm outperformed all other machine learning classifiers, achieving the highest AUC of 0.870 (95% CI, 0.817–0.922) and 0.791 (95% CI, 0.700–0.881) in both TT and ETV cohorts (Figure 3D and Table S5). Calibration curves showed good agreement between predicted and observed recurrence probabilities, and decision curve analysis demonstrated higher net clinical benefit than treat‐all or treat‐none strategies in both cohorts (Figure S3B,C).

### 2.3 SrMRI Model‐Derived TSR‐Radscore–Based Recurrence Prediction Model

We first evaluated the prognostic value of the pathologically assessed TSR. High‐TSR patients had significantly shorter recurrence‐free survival (RFS) than those with low TSR, with median RFS of 15.2 vs. 28.3 months (HR = 1.57, *p* = 0.029) in TT and 6.7 vs. 14.5 months (HR = 2.69, *p* = 0.002) in ETV cohort (Figure 3E,F). Next, we examined the recurrence‐predictive performance of the srMRI model‐derived TSR‐radscore. Table S6 summarizes the results of uni‐ and multivariable Cox regression of the srMRI model‐derived TSR‐radscore and clinicopathological variables based on the dataset of the TT cohort. The forest plot showed that the tumor size (HR = 1.38, *p* < 0.001), pN stage (HR = 1.52, *p* = 0.033), PFI (HR = 1.72, *p* = 0.028), and TSR‐radscore (HR = 3.12, *p* = 0.011) were independently associated with recurrence (Figure 4A).

**FIGURE 4:**
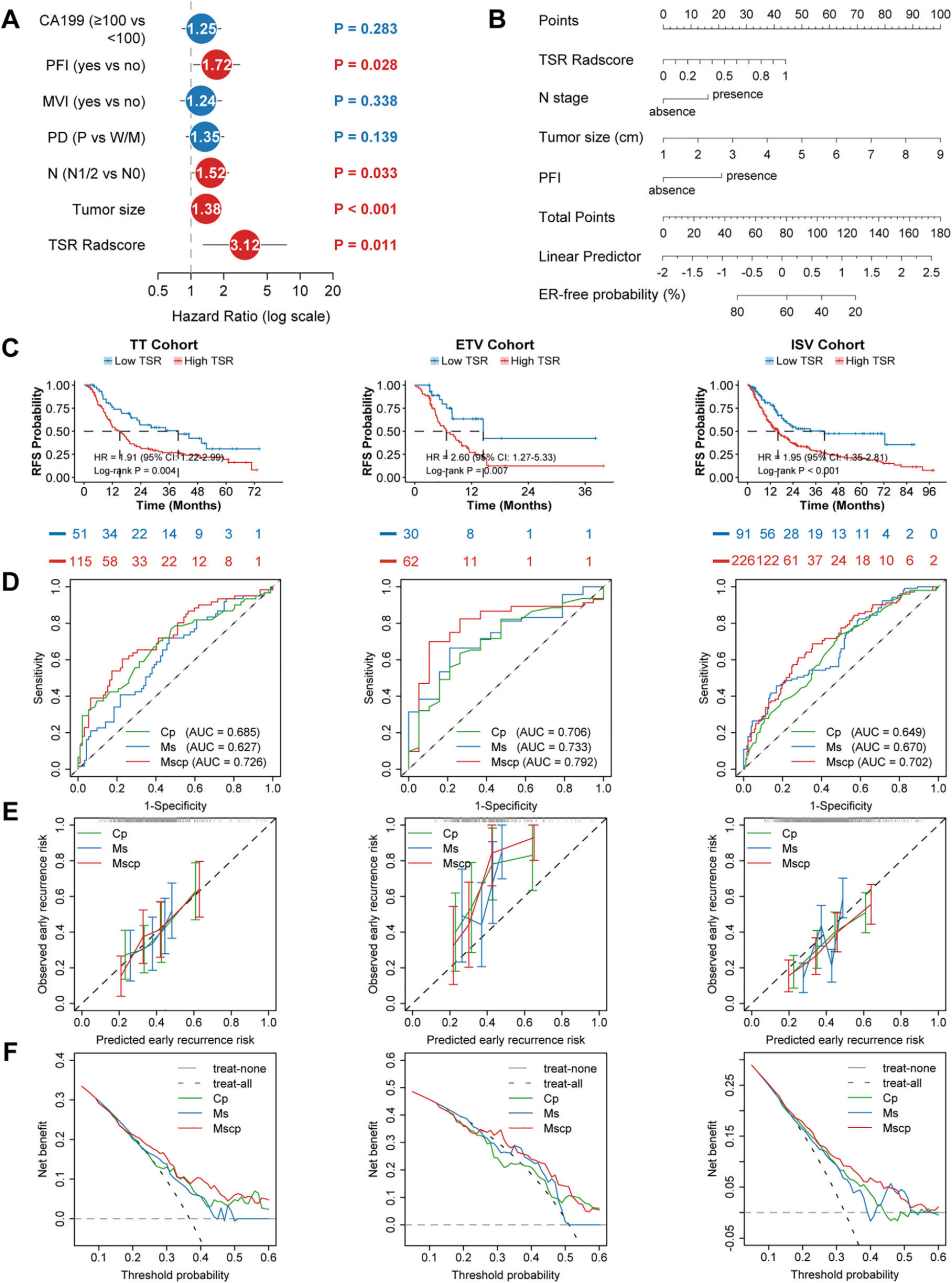
Prognostic performance and validation of the TSR‐guided srMRI model: (A) Multivariable Cox regression identifying independent predictors of RFS. The TSR‐radscore remained a significant prognostic factor after adjustment for clinicopathologic variables. (B) Nomogram incorporating TSR‐radscore, N stage, tumor size, and PFI for individualized ER prediction. (C) Kaplan–Meier curves showing significantly poorer RFS in high‐TSR patients across the TT, ETV, and ISV cohorts. (D) ROC curves for ER prediction comparing the clinical‐pathologic model (Cp), the srMRI‐derived TSR‐radscore model (Ms), and the combined model (Mscp). (E) Calibration curves demonstrating good agreement between predicted and observed ER risk. (F) Decision curve analysis showing that the integrated Mscp model provides the greatest net clinical benefit across threshold probabilities.

Based on four independent risk factors, a nomogram was constructed to predict ER (Figure 4B). Then, patients were stratified into high‐TSR (H‐TSR) and low‐TSR (L‐TSR) groups based on the optimal TSR‐radscore cutoff value of 0.612 determined in the TT cohort. Kaplan–Meier analysis demonstrated that H‐TSR patients had significantly worse RFS than those with low TSR across the TT, ETV, and ISV cohorts (HR: 1.91–2.60, all log‐rank *p* < 0.05; Figure 4C). ROC analyses for ER prediction showed that Mscp model (incorporating TSR‐radscore and clinicopathological parameters) consistently achieved higher AUC values than models with Cp or TSR‐radscore (Ms) alone, with AUCs of 0.726, 0.792, and 0.702 across three cohorts, respectively (Figure 4D). Calibration curves showed good agreement between predicted and observed recurrence risks across cohorts, and DCA indicated superior net clinical benefit of the Mscp model compared with Cp or Ms alone (Figure 4E,F).

### 2.4 Clinical Utility of srMRI‐Derived TSR in the LAPC Cohort

In the LAPC cohort, srMRI‐derived TSR stratification was associated with distinct treatment responses. H‐TSR patients exhibited significantly fewer progressive disease (PD) events than L‐TSR patients (*p* = 0.025; Figure 5A). Consistently, the L‐TSR group showed shorter PFS and a higher risk of progression (HR = 1.78, log‐rank *p* = 0.042), with a similar pattern observed for overall survival (OS) (HR = 2.13, log‐rank *p* = 0.045) (Figure 5B,C).

**FIGURE 5:**
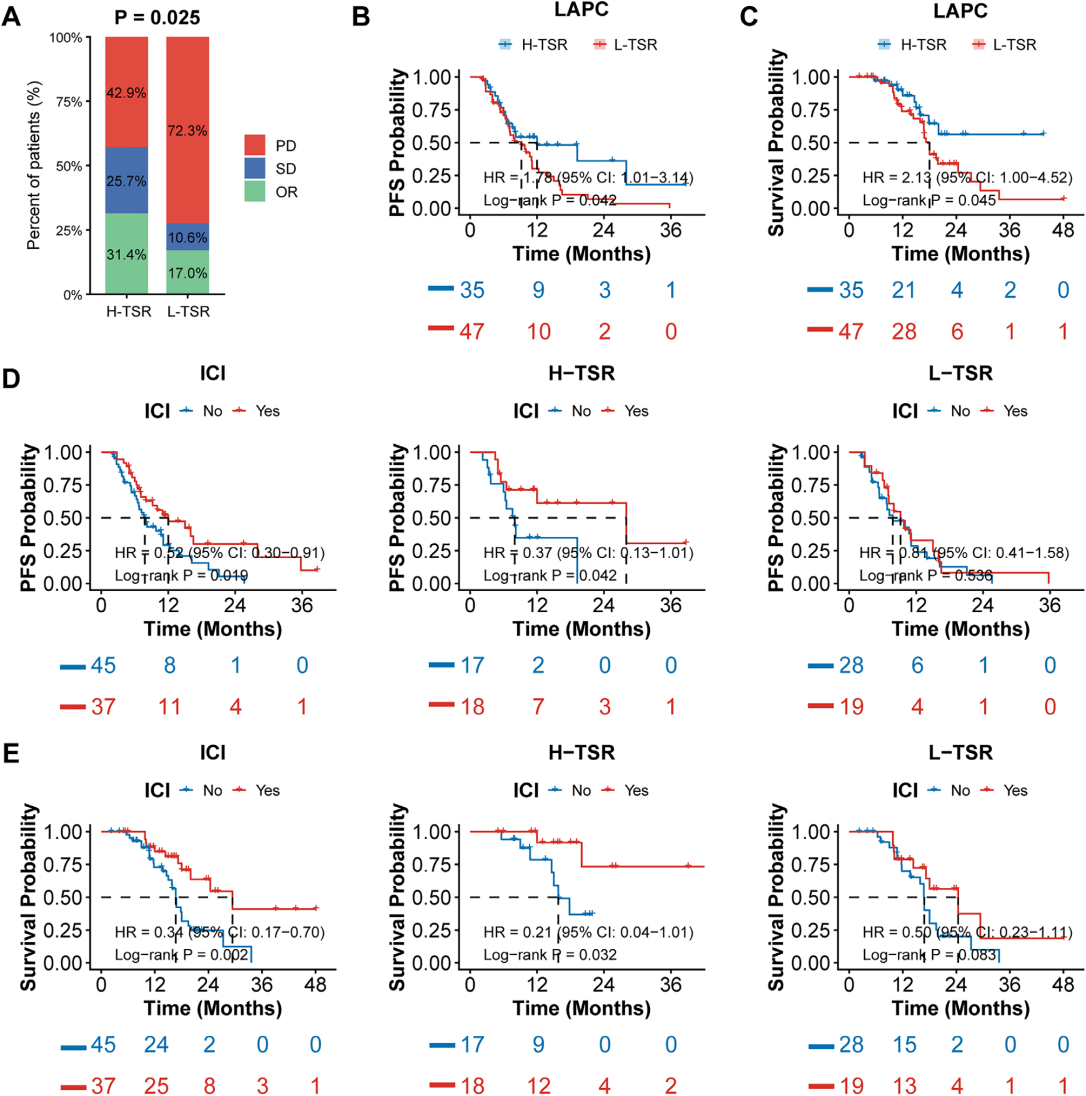
Association of srMRI model‐derived TSR phenotype with treatment response and survival outcomes in the LAPC cohort: (A) Distribution of treatment response categories (objective response, stable disease, and progressive disease) between H‐TSR and L‐TSR patients, showing significantly lower progressive disease rates in the high‐TSR group. (B) PFS curves comparing H‐TSR and L‐TSR subgroups in the LAPC cohort. (C) Overall survival curves showing significantly better outcomes in high‐TSR patients. (D) PFS stratified by immune checkpoint inhibitor (ICI) treatment in the overall LAPC cohort (left), H‐TSR subgroup (middle), and L‐TSR subgroup (right). (E) Overall survival stratified by ICI treatment in the overall cohort (left), H‐TSR subgroup (middle), and L‐TSR subgroup (right). Kaplan‐Meier curves were compared using two‐sided log‐rank tests, and HRs with 95% CIs were estimated using Cox proportional hazards models.

In the overall LAPC cohort, ICI‐based combination therapy was associated with improved PFS (HR = 0.52) and OS (HR = 0.34) compared with chemotherapy alone (Figure 5D,E), with individual tumor responses and chemotherapy backbone‐specific outcomes further detailed in Figure S4A,B and Table S7, respectively. Similar associations were observed in the IPTW‐weighted sensitivity analysis after balancing baseline characteristics (all absolute standardized mean differences < 0.1), with weighted hazard ratios of 0.47 for OS and 0.59 for PFS (Table S8). In subgroup analyses, the association between ICI‐based combination therapy and clinical outcomes was primarily observed in the H‐TSR subgroup (PFS:HR = 0.37; OS:HR = 0.21), whereas no comparable association was observed in the L‐TSR subgroup (Figure 5D,E).

### 2.5 Bulk Transcriptomic Characterization of the srMRI Model‐Derived TSR Phenotype

To explore the biological features associated with the srMRI‐derived TSR phenotype, patients in the BVT cohort were stratified into H‐TSR (*n* = 30) and L‐TSR (*n* = 38) groups according to the srMRI‐derived TSR phenotype, and their transcriptomic profiles and tumor microenvironment characteristics were subsequently compared. ESTIMATE analysis revealed significant differences in stromal abundance between the two groups (Figure 6A). L‐TSR tumors showed higher stromal and ESTIMATE scores but lower tumor purity than H‐TSR tumors, consistent with the histopathological definition of TSR. TSR‐radscores were negatively correlated with stromal‐related transcriptional programs, including ECM structural signatures, epithelial–mesenchymal transition (EMT), and transforming growth factor‐β (TGF‐β) signaling (Figure 6B), which were also significantly lower in H‐TSR tumors (Figure S5A). Immune deconvolution further supported reduced stromal and immune infiltration in H‐TSR tumors (Figure S5B). Consistent patterns were observed across multiple estimation algorithms (Figure 6C).

**FIGURE 6:**
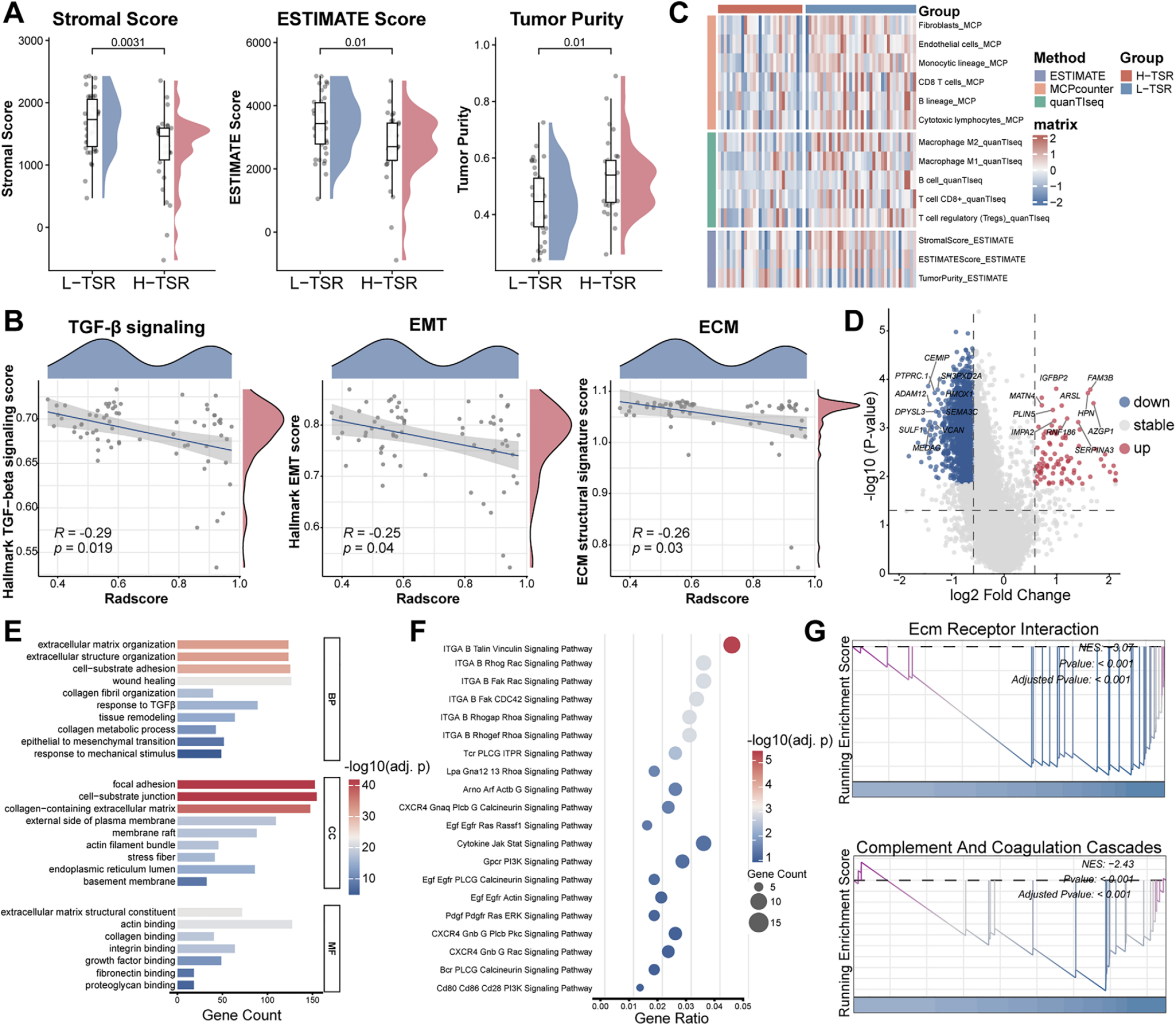
Bulk transcriptomic characterization of srMRI‐derived TSR phenotypes: (A) ESTIMATE analysis comparing stromal score, ESTIMATE score, and tumor purity between L‐TSR and H‐TSR tumors. (B) Correlation between TSR‐radscore and stromal‐related transcriptional programs (TGF‐β signaling, EMT, and ECM signatures). (C) Heatmap of stromal and immune‐related signatures estimated by multiple deconvolution methods across TSR subtypes. (D) Volcano plot showing differentially expressed genes between H‐TSR and L‐TSR tumors. (E) GO enrichment analysis highlighting ECM‐related biological processes. (F) Pathway enrichment analysis showing integrin‐ and cytoskeleton‐related signaling. (G) GSEA showing enrichment of ECM‐receptor interaction and complement/coagulation pathways in L‐TSR tumors.

Differential expression analysis identified distinct transcriptional patterns between TSR phenotypes, with several microenvironment‐related genes (e.g., *VCAN*, *SEMA3C*, and *ADAM12*) enriched in L‐TSR tumors, consistent with their stroma‐enriched context (Figure 6D). Functional enrichment analyses revealed that differentially expressed genes between TSR phenotypes were predominantly associated with ECM organization, collagen fibril formation, and cell–substrate adhesion (Figure 6E). Pathway analysis further highlighted integrin‐ and cytoskeleton‐related signaling pathways involved in matrix interaction (Figure 6F). Gene set enrichment analysis further showed enrichment of the ECM–receptor interaction and complement/coagulation pathways in L‐TSR tumors (Figure 6G).

### 2.6 Single‐Cell Characterization of TSR‐Associated Microenvironmental Heterogeneity

To further explore the cellular context underlying srMRI‐derived TSR phenotypes, single‐cell RNA sequencing was performed on 12 treatment‐naïve PDAC specimens, including seven H‐TSR and five L‐TSR tumors classified according to the srMRI‐derived TSR phenotype, yielding 147,669 cells across ten major cell populations (Figure 7A–D). Consistent with the bulk transcriptomic findings, srMRI‐derived L‐TSR tumors exhibited a higher proportion of fibroblasts, whereas epithelial and immune cell fractions were relatively increased in H‐TSR tumors.

**FIGURE 7:**
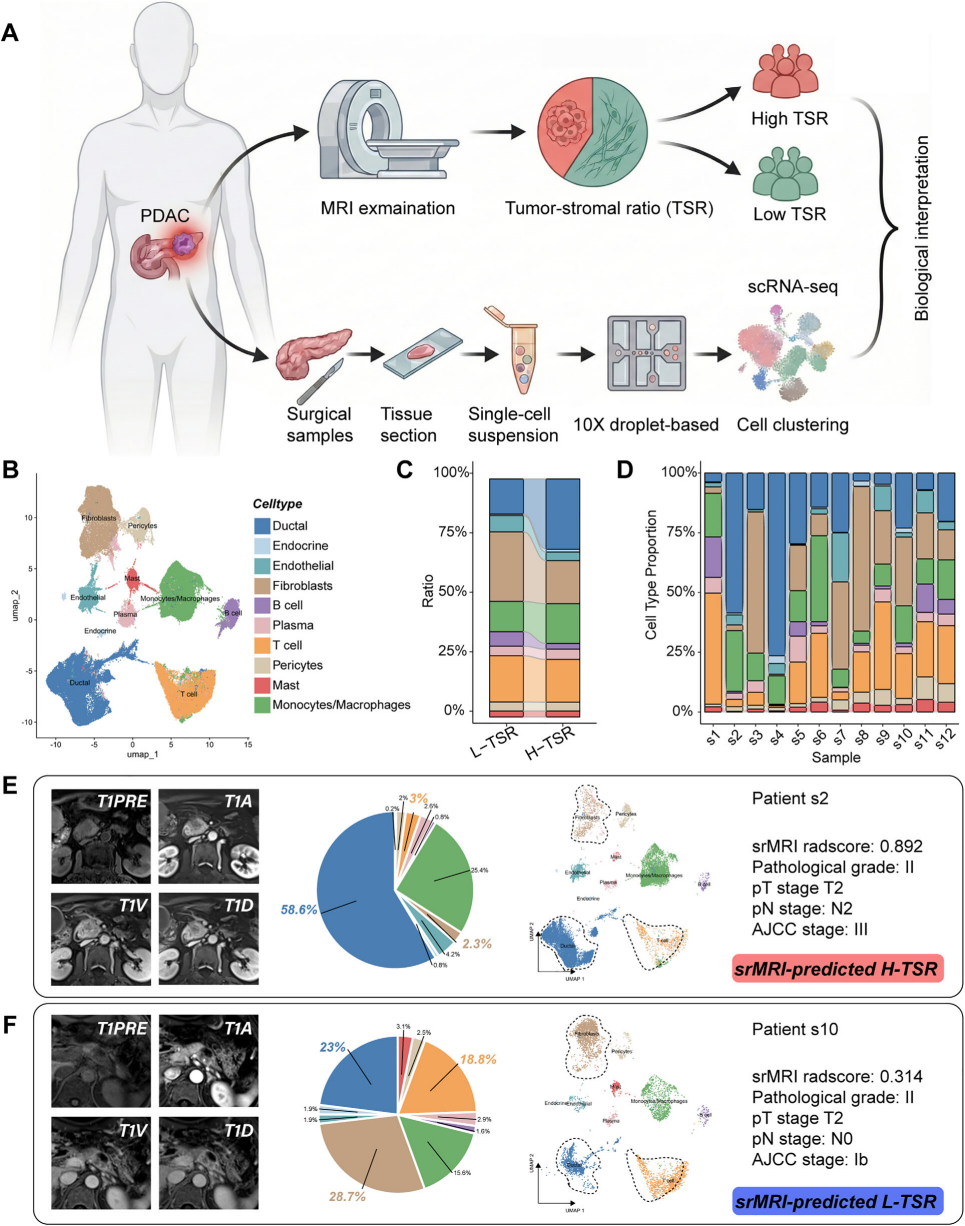
Cellular landscape underlying srMRI‐derived TSR phenotypes: (A) Study workflow integrating srMRI‐based TSR stratification with single‐cell transcriptomic profiling of treatment‐naïve PDAC specimens. (B) UMAP visualization of single‐cell transcriptomes from 12 PDAC tumors, resolving ten major cell populations. (C) Comparison of overall cell‐type composition between L‐TSR and H‐TSR tumors. (D) Stacked bar plot showing the proportion of major cell types across individual PDAC samples. (E,F) Representative case of srMRI‐predicted H‐TSR (E) and L‐TSR tumor (F) with corresponding MRI images, cell‐type composition, and UMAP visualization.

Further analysis of fibroblast subsets resolved eight CAF states, including LRRC15^+^ CAFs, inflammatory CAFs (iCAFs), apCAF‐like CAFs, quiescent CAFs, stress‐response CAFs, ion‐signaling CAFs, immediate‐early CAFs, and hCAFs (Figure S6A–C). Among these, LRRC15^+^ CAFs were more abundant in L‐TSR tumors (31.7% vs 15.2%), consistent with the stroma‐rich phenotype of this group, whereas ion‐signaling CAFs were relatively enriched in H‐TSR tumors (28.0% vs. 11.4%), suggesting differences in stromal activation states between TSR phenotypes (Figure S6D). Analysis of T‐cell subsets further indicated differences in T‐cell states between TSR phenotypes, with effector T cells more abundant in L‐TSR tumors (16.4% vs. 8.8%), whereas exhausted CD8^+^ T cells (Tex) were relatively enriched in H‐TSR tumors (27.2% vs. 8.6%) (Figure S6E–H). Together, these findings suggest that srMRI‐derived TSR phenotypes are associated with coordinated variations in stromal composition and T‐cell functional states within the PDAC microenvironment, potentially contributing to the distinct prognostic patterns and treatment responses observed between TSR subtypes.

## 3 Discussion

In this study, we demonstrated that a TSR‐guided srMRI radiomic model can noninvasively characterize stromal status and robustly stratify recurrence risk in PDAC. The srMRI‐derived TSR‐radscore remained an independent prognostic factor across three independent cohorts and further improved recurrence prediction when integrated with key clinicopathological variables (Mscp model). Notably, its relevance extended beyond resectable disease to the LAPC cohort, where the srMRI‐predicted TSR was associated with distinct therapeutic outcomes across chemotherapy and chemo‐immunotherapy regimens. Transcriptomic analyses provided biological support for these imaging findings. Bulk RNA sequencing revealed enrichment of ECM‐related transcriptional programs in L‐TSR tumors, whereas single‐cell profiling showed differences in stromal and immune cell states between TSR phenotypes.

ER following surgical resection remains a major challenge in PDAC and is increasingly understood as a manifestation of stromal remodeling and tumor–immune dysregulation within the tumor microenvironment [19, 20]. Moreover, these stromal and immune processes are simultaneously linked to therapeutic vulnerability in advanced disease, where response to chemotherapy and chemo‐immunotherapy varies markedly among patients. In this context, the TSR has emerged as a biologically grounded indicator reflecting both the risk of early metastatic spread and the therapeutic responsiveness of the tumor‐stroma ecosystem [21, 22]. Several studies have reported associations between TSR and both recurrence risk and chemotherapy response [23, 24]. However, current TSR assessment relies primarily on postoperative histopathology or limited biopsy material, which cannot adequately capture spatial stromal phenotyping or support pre‐treatment decision‐making. These limitations highlight the need for noninvasive approaches capable of quantifying stromal features in vivo, thereby enabling both recurrence risk assessment and treatment‐response stratification in clinical practice [25].

Following previous studies, a cutoff value of 50% was used to define TSR status, dividing patients into high‐TSR and low‐TSR groups [26, 27, 28]. Consistent with prior reports, patients with high TSR exhibited significantly shorter RFS in both the TT and ETV cohorts, supporting the prognostic relevance of stromal composition in PDAC [18]. Using multiparametric srMRI, we developed a TSR‐guided radiomics signature composed of 30 features that demonstrated robust predictive performance for TSR status. Compared with previously reported CT‐based TSR radiomics models, which typically achieved AUCs ranging from approximately 0.63 to 0.75, the srMRI‐based model showed improved performance [10, 17]. This advantage may be attributable to the superior soft‐tissue contrast of MRI and the complementary biological information captured by multiple MRI sequences, enabling more accurate noninvasive assessment of whole‐tumor stromal phenotypes.

Beyond TSR classification, the imaging‐derived TSR‐radscore demonstrated prognostic value across three cohorts, with H‐TSR patients showing significantly shorter RFS, in line with reports from other malignancies linking TSR‐based radiomic scores to metastatic risk [16]. Integrating the TSR‐radscore with established clinicopathological variables such as tumor size, pN stage, and PFI further improved predictive performance in the Mscp model, suggesting that imaging‐derived stromal features provide complementary prognostic information beyond conventional risk factors [29, 30, 31]. Importantly, the Mscp model maintained stable performance in the independent ISV cohort, indicating that the srMRI‐derived TSR biomarker remains generalizable across patient populations and may serve as a noninvasive surrogate of stromal status for ER risk stratification in PDAC.

In addition, the clinical relevance of the srMRI‐derived TSR extended beyond resectable disease. In the LAPC cohort, TSR stratification was associated with differential therapeutic outcomes, with the H‐TSR subgroup showing more favorable responses to chemotherapy and an exploratory association with ICI‐based combination therapy outcomes [32]. Together, these findings suggest that the TSR‐radscore captures tumor‐stromal features that remain clinically informative across different PDAC patient populations, supporting its potential role as both a prognostic indicator and a candidate biomarker for treatment stratification.

To further investigate the biological basis underlying the imaging‐derived TSR phenotype, we performed transcriptomic analyses in the BV cohort. Bulk RNA sequencing revealed that L‐TSR tumors exhibited higher stromal scores and enrichment of ECM‐related programs and CAF‐associated signatures, including EMT and TGF‐β signaling, supporting the stroma‐rich phenotype inferred from imaging. These findings provide a pathological rationale for srMRI‐derived TSR as a non‐invasive marker of stromal remodeling [33]. Given the link between stromal remodeling and therapeutic resistance, srMRI‐derived TSR may have utility for treatment stratification, although its predictive value requires prospective validation [34].

Single‐cell transcriptomic analysis further provided cellular‐level insights into these differences. Consistent with the bulk findings, L‐TSR tumors contained a higher proportion of fibroblasts, whereas epithelial and immune cell fractions were relatively increased in H‐TSR tumors. Within the fibroblast compartment, LRRC15^+^ CAFs were more abundant in L‐TSR tumors, consistent with the stromal‐dominant phenotype identified by the radiomic model, whereas ion‐signaling CAFs were relatively enriched in H‐TSR tumors, suggesting distinct stromal activation states between TSR phenotypes [35, 36]. Analysis of T‐cell subsets further indicated differences in immune states between TSR subtypes, with effector T cells more prevalent in L‐TSR tumors and exhausted CD8^+^ T cells relatively enriched in H‐TSR tumors. These observations suggest that H‐TSR tumors represent a distinct stromal‐immune state that may contribute to ER while remaining more susceptible to systemic therapies, consistent with recent single‐cell studies demonstrating that distinct CAF states influence tumor progression and immunotherapy response in PDAC [37, 38].

This study has several limitations. First, although the study incorporated multiple independent cohorts from different centers, the sample size of the TSR training cohort remained moderate, and the retrospective nature of the analysis may introduce potential selection bias. Validation in larger and more diverse populations will therefore be necessary to further confirm the generalizability of the model. In addition, given the limited subgroup size, retrospective design, and wide confidence intervals, the treatment‐stratification findings in the ICI subgroup of the LAPC cohort should be considered exploratory and require prospective validation. Second, TSR was assessed in a representative tumor region on whole‐slide images of surgical resection specimens, which may not fully reflect the spatial heterogeneity of PDAC. Automated whole‐slide image‐based TSR quantification may provide a more comprehensive pathological reference in future studies. Third, although we explored the biological basis of the imaging‐derived TSR phenotype using bulk and single‐cell transcriptomic analyses, these findings remain descriptive and correlative, requiring validation in larger cohorts and further functional studies. In addition, protein‐level validation using immunohistochemistry or multiplex immunofluorescence was unavailable in the current retrospective cohort and warrants further validation in future studies. Finally, although srMRI provides improved soft‐tissue contrast for stromal characterization, its broader clinical application may be limited by variability in imaging protocols, the lack of standardized acquisition strategies across institutions, and the need for further evaluation of the incremental value of SR preprocessing over native MRI.

In conclusion, we developed and validated a srMRI‐based model for preoperative assessment of the TSR in PDAC. The srMRI‐derived TSR‐radscore accurately predicted pathological TSR and provided independent prognostic value for ER, while also showing potential for treatment response stratification. Bulk and single‐cell transcriptomic analyses provided biological context for the imaging‐derived phenotype. These results support srMRI‐based TSR as a noninvasive indicator of stromal status for recurrence‐risk assessment and systemic treatment decision‐making in PDAC.

## 4 Materials and Methods

### 4.1 Patients

This study was approved by the Institutional Review Board of our institution (B2025‐522) and informed consent was obtained in the study.

Patients with PDAC and available TSR data from archived paraffin‐embedded specimens at a national medical center (Center 1) between May 2016 and December 2020 were screened to construct the TSR training (TT) cohort. An external TSR validation (ETV) cohort was assembled from two independent centers (BBMU [Center2] and ZSXMFD [Center3]) between January 2019 and September 2023. For clinical utility evaluation, an ISV cohort included patients with surgically confirmed PDAC who underwent preoperative MRI and curative‐intent resection at Center 1 (January 2015–December 2022). Cases in the TT cohort were excluded from the ISV to prevent overlap. Moreover, patients with locally advanced pancreatic cancer (LAPC) receiving chemotherapy or chemoimmunotherapy at Center 1 (June 2019–May 2023, *n* = 82) were enrolled as a treatment validation cohort. Dataset allocation was performed at the patient level, with all MRI sequences from the same patient assigned to the same cohort.

Furthermore, a biological validation (BV) cohort of treatment‐naïve PDAC patients (October 2023–September 2025) was established for multi‐omics BV. This cohort comprised the BV‐Transcriptome (BVT) sub‐cohort for bulk RNA sequencing (*n* = 68) and the BV‐SingleCell (BVS) sub‐cohort for single‐cell RNA sequencing (*n* = 12) to explore the molecular and cellular basis of the srMRI model‐derived phenotypes. Cohort‐specific eligibility criteria, study design, and clinicopathological characteristics are detailed in Supporting Appendix S1.

### 4.2 Clinical–Radiological and Pathological Data

Clinical, radiological, and pathological data were systematically retrieved. Clinical variables encompassed age, sex, body mass index (BMI), diabetes mellitus (DM), hypertension (HBP), CA19‐9, total bilirubin, and albumin levels, alongside records of adjuvant chemotherapy. MRI‐based variables included tumor size and location. Pathology reports provided primary tumor (pT), lymph node (LN) involvement (pN), American Joint Committee on Cancer (AJCC) (eighth edition) staging, pathological differentiation (PD), lymphovascular invasion (PVI), perineural invasion (PNI), and peripancreatic fat invasion (PFI; defined as tumor extension beyond the pancreatic parenchyma into the surrounding adipose tissue). Full data‐extraction workflow is described in Supporting Appendix S1.

### 4.3 Follow‐Up Analysis

Postoperative follow‐up occurred every 3 months for 1 year and every 6 months thereafter. OS was calculated from surgery or initiation of chemotherapy to death or last follow‐up. Progression‐free survival (PFS) was defined as the interval from chemotherapy or chemoimmunotherapy initiation to imaging‐confirmed progression (RECIST v1.1), initiation of another antitumor therapy, or death. RFS applied to resected patients only and was calculated from surgery to recurrence, death, or censoring. Patients without events by December 2024 were censored.

### 4.4 TSR Assessment

TSR evaluation was performed on digitized whole‐slide images of H&E‐stained sections derived from formalin‐fixed paraffin‐embedded surgical resection specimens. Following established criteria, the whole‐slide image was reviewed to identify the most invasive tumor area, where the relative proportion of stromal and tumor cells was estimated at ×100 magnification [39, 40]. TSR was assessed as the tumor‐to‐stromal proportion on H&E‐stained sections, and tumors were classified as low‐TSR (TSR ≤ 1) or high‐TSR (TSR > 1), corresponding to a 50% stromal cutoff. TSR evaluation was independently performed by two pathologists with experience in pancreatic pathology (Reader 1 and Reader 2, with 7 and 12 years of diagnostic experience, respectively), blinded to clinical–pathological data. In cases of disagreement, a third senior pathologist reviewed the slides for consensus. Interobserver agreement was evaluated using Cohen's *κ* statistics to ensure scoring reproducibility.

### 4.5 MRI Protocols

MRI examinations were performed on 1.5‐T and 3.0‐T scanners across participating centers during the study period. The standardized protocol included six sequences: axial T2‐weighted imaging (T2), diffusion‐weighted imaging (DWI), unenhanced T1‐weighted imaging (T1PRE), and three contrast‐enhanced T1‐weighted phases—arterial (T1A), portal venous (T1V), and delayed (T1D). Detailed acquisition parameters are provided in Supporting Appendix S2 and Table S1.

### 4.6 Super‐Resolution MRI‐Based Radiomic Analysis

#### 4.6.1 Tumor Processing

Six MRI sequences, including T2, DWI, T1PRE, T1A, T1V, and T1D, were manually segmented using ITK‐SNAP (version 4.0.2) and utilized for subsequent analyses. Super‐resolution reconstruction and imaging harmonization were performed prior to feature extraction. The full segmentation and preprocessing workflows are described in Supporting Appendix S3.1 and S3.2.

### 4.7 Feature Extraction and Selection

Handcrafted radiomic features were extracted from preoperative multiparametric MRI, encompassing six sequences (T2WI, DWI, T1PRE, T1A, T1V, and T1D). These features quantified tumor morphology, signal intensity distribution, and textural heterogeneity within the segmented volumes. To ensure robustness, features with low reproducibility or high inter‐feature redundancy were excluded, and the most informative predictors were selected using least absolute shrinkage and selection operator (LASSO) regression. The radiomic score (TSR‐radscore) was subsequently constructed using the selected features. Detailed extraction workflows are provided in Supporting Appendix S3.3.

### 4.8 Bulk RNA Sequencing and Transcriptomic Analysis

Bulk RNA sequencing was performed on tumor specimens from the BV cohort. Based on srMRI‐derived TSR, samples were stratified into high‐TSR (H‐TSR) and low‐TSR (L‐TSR) groups for transcriptomic comparison. Sequencing libraries were constructed following poly(A) mRNA enrichment and sequenced on an Illumina platform. Gene expression levels were quantified and normalized, and log‐transformed expression matrices were used for downstream analyses. Differential gene expression analysis, gene set enrichment analysis (GSEA), Gene Ontology (GO), Kyoto Encyclopedia of Genes and Genomes (KEGG) pathway enrichment, and single‐sample gene set enrichment analysis (ssGSEA) were performed to characterize TSR‐associated transcriptomic programs. Detailed analytical procedures and parameter settings are provided in Supporting Appendix S4.

### 4.9 Preparation and Analysis of Single‐Cell RNA Sequencing

To investigate TSR‐associated stromal and immune heterogeneity within the tumor microenvironment (TME), single‐cell RNA sequencing (scRNA‐seq) was performed on 12 freshly resected PDAC specimens from the BV cohort. Samples were stratified into H‐TSR and L‐TSR groups according to the srMRI‐derived TSR phenotype.

Following tissue dissociation and single‐cell suspension preparation, cDNA libraries were constructed and sequenced using the 10X Genomics platform. Subsequent computational processing involved standard quality control, doublet removal, normalization, and dimensionality reduction, followed by unsupervised clustering and cell type annotation. Comparative analyses between H‐TSR and L‐TSR groups were then performed to delineate distinct cellular compositions and transcriptional programs. Detailed analytical procedures are provided in Supporting Appendix S5.

### 4.10 Statistical Analysis

Baseline characteristics were compared using the Student's *t*‐test or Mann–Whitney *U*‐test for continuous variables and the *χ* ^2^ or Fisher's exact test for categorical variables. Model discrimination was assessed by the area under the ROC curve (AUC) with 95% confidence intervals (CIs). Sensitivity, specificity, accuracy, PPV, NPV, and F1‐score were calculated, with internal robustness evaluated by five‐fold cross‐validation and external validity confirmed in ETV cohort. The srMRI model‐derived TSR‐radscore was then analyzed as a TSR‐guided imaging marker for recurrence. Univariable Cox regression was performed, and variables with *p* < 0.05 were entered into a multivariable Cox model using backward stepwise selection to identify independent predictors of recurrence. Additionally, Kaplan–Meier curves were generated for patients stratified into high‐ and low‐TSR‐radscore groups using cutoffs derived from pathological TSR, with the LAPC cohort further validating its prognostic relevance and treatment‐response stratification. Predictive performance for ER was further assessed using ROC curves, calibration, and decision‐curve analysis (DCA). Treatment‐selection bias in the LAPC cohort was addressed by stabilized inverse‐probability‐of‐treatment weighting (IPTW), with covariate balance assessed by standardized mean differences. A two‐sided *p* value < 0.05 was considered statistically significant. All analyses were conducted using R software (version 4.4.1).

## Author Contributions

X.W., Z.X., and H.L. contributed equally to this work. X.W., H.L., M.H., S.F., Q.T., J.H., Q.Z., J.Z., Y.X., and H.S. contributed to the conception and design of the study. X.W., H.L., M.H., Q.T., L.L., S.F., D.L., J.H., Q.Z., Y.X., and H.S. were responsible for data acquisition, clinical information curation, and quality control. L.L., D. L., S.F., M.Z., Q.Z., J.Z., Y.X., and H.S. performed data analysis, statistical modeling, and interpretation of the results. M.Z. and Q.Z. provided administrative support for the study. X.W., H.L., Z.X., and H.S. drafted the initial manuscript. Q.Z., M.H., Y.X., and H.S. critically revised the manuscript for important intellectual content and supervised the overall study. Q.Z., M.H., Y.X., and H.S. served as guarantors of the integrity of the entire work. All authors reviewed and approved the final version of the manuscript.

## Funding

This work was supported by the Shanghai Municipal Health Commission Health Industry Clinical Research Special Program (Youth Project) (20244Y0003), 2025 Open Project Approval Form of the Anhui Key Laboratory of Digital Medicine and Intelligent Health (Bengbu Medical University) (AHCM2025Z001), the Anhui Provincial Medical Imaging Discipline Leader Cultivation Project (DTR2024028); the Bengbu Medical University Key Support and Cultivation Discipline Area Special Project (Quantum Medicine Project) (2024bypy017); the Longhu Talent Project of Bengbu Medical University (LH250302002); and the Jianghuai Talent Development Program—Distinguished Project.

## Conflicts of Interest

The authors declare no conflicts of interest.

## Ethics Approval

This study was approved by the Institutional Review Board of Zhongshan Hospital, Fudan University (Approval No. B2025‐522) and informed consent was obtained from all participants in the study.

## Supporting information



Supporting information

## Data Availability

The datasets analyzed in this study are available from the corresponding author upon reasonable request and completion of a relevant research agreement. Summary statistics for all cohorts are provided in the supporting tables. The raw bulk RNA‐seq and single‐cell RNA‐seq data generated in this study have been deposited in GSA‐Human under accession numbers HRA019322 and HRA019286, respectively (BioProject: PRJCA067503). All data preprocessing, radiomic feature extraction, and model development were performed on the OmniMedAI platform (https://www.omnimai.com), and the associated project source code has been open‐sourced in the GitHub repository (https://github.com/OminiMedAI/pdac_tsr_srmri).
