# Supplementary material for: Super‐Resolution MRI–Derived Tumor‐Stroma Ratio Phenotyping for Prognostic and Therapeutic Stratification in Pancreatic Ductal Adenocarcinoma
Source: MedComm (2020). 2026 Oct 9;7(10):e71039. doi: 10.1002/mco2.71039 (PMC13650606; doi:10.1002/mco2.71039)
Supplement: Supplementary file 1 [file MCO2-7-e71039-s001.docx]

**Supplemental information**

**Super-Resolution MRI-Derived Tumor-Stroma Ratio Phenotyping for Prognostic and Therapeutic Stratification in Pancreatic Ductal Adenocarcinoma**

**Running title:** srMRI-derived TSR phenotyping in PDAC

Xingxing Wang,^1^^#^, Zongyu Xie,^2,10#^, Hanxiao Li,^3#^, Qiying Tang,^4^, Jianjun Zhou,^4^, Jingcheng Huang,^2^, Shiwu Fang,^2^, Dalong Li,^5^, Lei Li,^6^, Mengsu Zeng,^3^, Minping Hong,^7*^, Qiuyang Zhu,^8*^, Yaolin Xu,^9*^, Haitao Sun,^3,4,10*^

^#^ Xingxing Wang, Zongyu Xie, and Hanxiao Li contributed equally to this work.

^*^Qiuyang Zhu, Minping Hong, Yaolin Xu and Haitao Sun are co-corresponding authors.

a

1. Department of Pathology, Zhongshan Hospital, Fudan University, No. 180 Fenglin Road, Xuhui District, Shanghai 200032, China

2. Department of Radiology, The First Affiliated Hospital of Bengbu Medical University, Bengbu, China

3. Department of Radiology, Zhongshan Hospital, Fudan University, Shanghai Institute of Medical Imaging, No. 180 Fenglin Road, Xuhui District, Shanghai 200032, China

4. Department of Radiology, Zhongshan Hospital (Xiamen), Fudan University, Xiamen 361015, China

5. Department of Radiology, Second Affiliated Hospital of Bengbu Medical University, Bengbu, China

6. Department of Radiology, Fengyang County People’s Hospital, Chuzhou, China

7. Department of Radiology, Jiaxing Hospital of Traditional Chinese Medicine Affiliated to Zhejiang Chinese Medical University, Jiaxing, China

8. School of Instrument and Electronics, North University of China, Shanxi; Ceyear Technologies Co. Ltd., Shandong, China

9. Department of Pancreatic Surgery, Zhongshan Hospital, Fudan University, Shanghai, China. Cancer Center, Zhongshan Hospital, Fudan University, Shanghai, China.

10. Anhui Key Laboratory of Digital Medicine and Intelligent Health, Bengbu, China.

**Corresponding authors**

**Corresponding author**: Haitao Sun

**Telephone:** 86-021-64041990

**Email address:** sht1720@163.com

**Complete address:** Department of Radiology, Zhongshan Hospital, Fudan University, Shanghai Institute of Medical Imaging, No. 180 Fenglin Road, Xuhui District, Shanghai 200032, China

**Corresponding author**: Yaolin Xu

**Telephone:** 86-021-64041990

**Email address:** xuyaolin920816@gmail.com

**Complete address:** Department of Pancreatic Surgery, Zhongshan Hospital, Fudan University, Shanghai, China. Cancer Center, Zhongshan Hospital, Fudan University, Shanghai, China

**Corresponding author**: Qiuyang Zhu

**Email address:** zqyhit@qq.com

**Complete address:** School of Instrument and Electronics, North University of China, Shanxi; Ceyear Technologies Co. Ltd., Shandong, China

**Corresponding author**: Minping Hong

**Email address:** 15757338851@163.com

**Complete address:** Department of Radiology, Jiaxing Hospital of Traditional Chinese Medicine Affiliated to Zhejiang Chinese Medical University, Jiaxing, China

[Appendix S1	4](#_Toc238568682)

[S1.1 Detailed criteria for the TSR training (TT) and external TSR validation (ETV) cohorts	4](#_Toc238568683)

[S1.2 Detailed data for LAPC cohort	4](#_Toc238568684)

[S1.3 Detailed criteria for the Biological Validation (BV) cohort	5](#_Toc238568685)

[S1.4 Detailed Clinical and Radiological Data Analysis	5](#_Toc238568686)

[Appendix S2	6](#_Toc238568687)

[S2.1 Detailed Imaging Acquisition	6](#_Toc238568688)

[Appendix S3	6](#_Toc238568689)

[S3.1 Image Preprocessing	6](#_Toc238568690)

[S3.2 Tumor Segmentation and Super-Resolution MRI Reconstruction	6](#_Toc238568691)

[S3.3 Radiomic Feature Extraction	7](#_Toc238568692)

[Appendix S4	9](#_Toc238568693)

[Bulk RNA sequencing and transcriptomic analysis	9](#_Toc238568694)

[S4.1 RNA sequencing and data processing	9](#_Toc238568695)

[S4.2 Differential gene expression analysis	9](#_Toc238568696)

[S4.3 Functional enrichment analysis	9](#_Toc238568697)

[S4.4 Gene set enrichment analysis	10](#_Toc238568698)

[S4.5 Gene signature scoring	10](#_Toc238568699)

[S4.6 Tumor microenvironment estimation	10](#_Toc238568700)

[Appendix S5	11](#_Toc238568701)

[Preparation and analysis of single-cell RNA sequence	11](#_Toc238568702)

[S5.1 Preparation of Tissue	11](#_Toc238568703)

[S5.2 Single-cell library construction and sequencing workflow	11](#_Toc238568704)

[S5.3 Data processing, clustering, and cell-type annotation	11](#_Toc238568705)

[Supplementary table	13](#_Toc238568706)

[Table S1 MRI acquisition parameters across multi-centers	13](#_Toc238568707)

[Table S2 Patient demographics and clinicopathological variables across TSR subgroups	15](#_Toc238568708)

[Table S3 Characteristics in LAPC validation cohort stratified by srMRI-based model	18](#_Toc238568709)

[Table S4 Performance of srMRI Radiomics Models from Single Sequence	19](#_Toc238568710)

[Table S5 Performance of srMRI Model Using Different Machine Learning Algorithms for Predicting Tumor-Stroma Ratio	24](#_Toc238568711)

[Table S6 Uni- and Multivariate Regression of Independent Risk Factors for Recurrence	24](#_Toc238568712)

[Table S7. Treatment distribution and survival outcomes by chemotherapy backbone in the LAPC cohort	25](#_Toc238568713)

[Table S8 Baseline characteristics of the LAPC cohort by ICI treatment and covariate balance before and after IPTW	26](#_Toc238568714)

[Supplementary Figures	28](#_Toc238568715)

# Appendix S1

## S1.1 Detailed criteria for the TSR training (TT) and external TSR validation (ETV) cohorts

Patients were eligible for inclusion if they met the following criteria: (1) pathologically confirmed pancreatic ductal adenocarcinoma after surgical resection; (2) availability of complete clinical, imaging, and pathological data; and (3) no prior anticancer treatment such as chemotherapy or radiotherapy before surgery. Patients were excluded if they met any of the following conditions: (1) missing or poor-quality preoperative MRI imaging; (2) incomplete clinical or follow-up data; (3) presence of other synchronous malignant tumors; or (4) receipt of preoperative anticancer treatment. To avoid cohort overlap, cases included in the TT cohort were excluded from the independent surgical validation (ISV) cohort.

## S1.2 Detailed data for LAPC cohort

All patients in LAPC cohort had pathologically confirmed PDAC, diagnosed via fine-needle aspiration (FNA). In this cohort, chemotherapy regimens included AG (albumin-bound paclitaxel + gemcitabine) or AS (albumin-bound paclitaxel + S-1), S-1, gemcitabine, and GS (gemcitabine + S-1). A subset of patients additionally received anti-PD-1 therapy (tislelizumab, sintilimab, or pembrolizumab) according to routine clinical practice and multidisciplinary evaluation. Inclusion criteria were: (1) Pathologically confirmed diagnosis of PDAC; (2) Surgical assessment confirming LAPC, followed by chemotherapy alone or chemotherapy combined with immunotherapy; (3) Pre-treatment dynamic contrast-enhanced MRI; (4) Availability of comprehensive clinical data, including demographic information, laboratory results, treatment regimens and timelines, pathological findings, and follow-up data. Exclusion criteria were: (1) Prior receipt of other anti-tumor therapies before examination; (2) Absence of pre-treatment MRI; (3) Poor-quality imaging; (4) Lack of survival follow-up data.

## S1.3 Detailed criteria for the Biological Validation (BV) cohort

Patients included in the BV cohort underwent surgical resection for PDAC between October 2023 and September 2025. The BV cohort (n=80) comprised two sub-cohorts: the BV-Transcriptome (BVT) cohort (n=68) and the BV-SingleCell (BVS) cohort (n=12). Patients were eligible if they met the following criteria: (1) surgical-pathologically confirmed PDAC; (2) no prior anticancer treatment before surgery; (3) availability of preoperative contrast-enhanced MRI; and (4) availability of tumor specimens suitable for transcriptomic analysis, including bulk RNA sequencing or single-cell RNA sequencing. For all included patients, transcriptomic data were paired with the corresponding preoperative MRI, and patients were stratified according to the srMRI-derived TSR phenotype for subsequent transcriptomic analyses. Patients were excluded if they met any of the following conditions: (1) incomplete or poor-quality imaging data; (2) insufficient tumor tissue for molecular analysis; or (3) tumor samples failing quality assessment for bulk or single-cell RNA sequencing. Patients who met all eligibility criteria were finally included in the BV cohort.

## S1.4 Detailed Clinical and Radiological Data Analysis

Adjuvant chemotherapy regimen included Gemcitabine + Nab-Paclitaxel, FOLFIRINOX/mFOLFIRINOX regimen, or Gemcitabine + S-1/capecitabine.

For imaging interpretation, two abdominal radiologists with 9 and 12 years of experience independently re-examined all MRI scans in a blinded fashion, without access to clinical or pathological information. Imaging features were assessed separately by the two readers, and any discordant findings were adjudicated by a senior radiologist specializing in pancreatic MRI with over 30 years of experience, who rendered the final decision. Radiological variables were reassessed, including: (a) maximal axial tumor diameter, recorded as tumor size; and (b) anatomical tumor site, classified as pancreatic head/uncinate process versus body/tail.

# Appendix S2

## S2.1 Detailed Imaging Acquisition

All contrast-enhanced MRI examinations were performed on 1.5T or 3.0T systems, including MAGNETOM Aera, Prisma, and Amira (Siemens), Ingenia and Ingenia CX (Philips). Patients fasted for 6–8 hours prior to imaging. Gadolinium-based contrast agents—gadopentetate dimeglumine (Gd-DTPA; Magnevist), gadobenate dimeglumine, or gadoxetate disodium—were intravenously administered to reach the ascending aorta; Gd-DTPA was injected at a dose of 0.1 mmol/kg with a flow rate of 1.0–2.0 mL/s. The standardized MRI protocol included: (a) T2-weighted imaging; (b) diffusion-weighted imaging with b-values of 0 and 500 s/mm² or 0 and 800 s/mm²; (c) T1-weighted in-phase and out-of-phase imaging; and (d) dynamic contrast-enhanced T1-weighted imaging acquired in the precontrast, arterial (20–30 s), portal venous (60–70 s), and delayed (160–180 s) phases. Gadolinium-based contrast media, including gadopentetate dimeglumine (Gd-DTPA; Magnevist), gadobenate dimeglumine, and gadoxetate disodium, were intravenously injected to reach the ascending aorta. Gd-DTPA was administered at 0.1 mmol/kg with an injection rate of 1.0–2.0 mL/s.

# Appendix S3

## S3.1 Image Preprocessing

All MRI datasets underwent standardized preprocessing before feature extraction. N4 bias field correction was applied using ANTs to mitigate intensity non-uniformity across scanners. Each sequence was then resampled to an isotropic voxel size of 1 × 1 × 1 mm³, followed by gray-level discretization and z-score normalization. These steps ensured cross-patient and cross-sequence comparability and minimized variations attributable to acquisition differences.

## S3.2 Tumor Segmentation and Super-Resolution MRI Reconstruction

Tumor ROIs were manually delineated using ITK-SNAP (v4.0.2) by a radiologist with 10 years of abdominal imaging experience. Lesions with uncertain margins were reviewed and confirmed by a senior pancreatic imaging specialist (J.J.Z., >30 years of experience). To assess segmentation reproducibility, a second radiologist (H.T.S.) independently re-segmented MR images from 30 randomly selected patients after a two-month interval, blinded to the initial annotations. Radiomic features were extracted from each segmentation, and inter-observer and intra-observer intraclass correlation coefficients (ICCs) were calculated. Only features with ICC ≥ 0.80 in both assessments were retained for subsequent modeling to ensure robustness against operator-related variability.

Super-resolution reconstruction using a generative adversarial network was employed to improve the stability of both radiomic and deep learning-derived features by minimizing partial volume effects and enhancing image fidelity^1, 2^. This process provided a more robust computational basis for subsequent multimodal biomarker analyses in pancreatic cancer imaging. All multiparametric MRI sequences (T2WI, DWI, T1PRE, T1A, T1D, and T1V) underwent enhancement via a 4×Generative Adversarial Network for image super-resolution (SRGAN), trained on a dataset of 166 pancreatic MRI examinations. A reconstruction framework built on cascaded dense-residual modules with spectral normalization produced high-fidelity outputs, reaching an average PSNR of 50.65 ± 3.97 dB and an SSIM of 0.998 ± 0.003 on a separate validation dataset. After processing, the original clinical voxel dimensions (0.8-1.0 mm in-plane and 2.0-3.0 mm through-plane) were rendered into an isotropic grid of 0.2-0.25 mm³, which markedly reduced partial-volume blurring and enabled more faithful preservation of subtle parenchymal textures.

## S3.3 Radiomic Feature Extraction

Radiomic features were then derived from the three-dimensional tumor volumes using the PyRadiomics package (v3.1.0). In total, 1197 quantitative imaging features were generated per sequence. These consisted of 14 parameters characterizing volumetric shape and size, 234 first-order metrics describing the global distribution of signal intensities, and a series of higher-order textural features: 286 gray-level co-occurrence matrix (GLCM) features capturing spatial intensity co-dependence, 208 gray-level run-length matrix (GLRLM) features reflecting run-length variations, 208 gray-level size zone matrix (GLSZM) features summarizing homogeneous zone sizes, 182 gray-level dependence matrix (GLDM) features quantifying local dependence relationships, and 65 neighborhood gray-tone difference matrix (NGTDM) features evaluating tonal differences between adjacent voxels.

To assess feature robustness, both inter- and intra-observer ICCs were computed, and any feature with an ICC < 0.80 was excluded from further analysis. Radiomic feature batch effects across scanners were corrected using ComBat harmonization before feature selection. Redundancy was additionally reduced through Pearson correlation filtering, whereby one variable from each pair with r > 0.90 was removed. Subsequent feature selection in the training cohort was performed using LASSO regression with tenfold cross-validation. Feature selection was performed exclusively in the training cohort, whereas the validation cohorts were used solely for independent evaluation. The final radiomic signature (Radscore) was obtained from the probability outputs of an XGBoost classifier (hyperparameters: n_estimators = 100, max_depth = 4, learning_rate = 0.05, min_child_weight = 3, gamma = 0.05, subsample = 0.75, colsample_bytree = 0.70, reg_alpha = 0.05, and reg_lambda = 2.0). The optimal TSR-radscore cutoff (0.612) was determined in the training cohort and subsequently applied to all validation cohorts for patient stratification.

Subsequent data handling and model development were carried out on the OmniMedAI platform under Python 3.12.10. Statistical evaluation was performed with Statsmodels (v0.13.2), feature extraction with PyRadiomics (v3.1.0), and machine learning classifiers—including support vector machines (SVM), Logistic Regression (LR), Naive Bayes, K-Nearest Neighbors (KNN), Random Forest, ExtraTrees, and XGBoost—were implemented using Scikit-learn (v1.6.1).

**Reference**

1 E.C. de Farias, C. di Noia, C. Han, E, et al (2021) Rundo, Impact of GAN-based lesion-focused medical image super-resolution on the robustness of radiomic features, Sci. Rep. 11 (1) 21361.

2 J. Zhong, Y. Xia, Y. Chen, et al (2023) Deep learning image reconstruction algorithm reduces image noise while alters radiomics features in dual-energy CT in comparison with conventional iterative reconstruction algorithms: a phantom study, Eur. Radiol. 33 (2) 812–824.

# Appendix S4

## Bulk RNA sequencing and transcriptomic analysis

## S4.1 RNA sequencing and data processing

Total RNA was extracted from tumor tissues and subjected to library construction following standard protocols. Briefly, mRNA was enriched using poly-T oligo-attached magnetic beads, fragmented, and reverse-transcribed into cDNA. Sequencing libraries were generated through end repair, adapter ligation, and PCR amplification, followed by quality assessment using Qubit and Bioanalyzer. Qualified libraries were pooled and sequenced on an Illumina platform with paired-end reads.

Raw sequencing reads were processed using fastp to remove adapter sequences and low-quality reads. Clean reads were aligned to the human reference genome using HISAT2 (v2.2.1). Gene-level counts were obtained using featureCounts (v2.0.6) and normalized as FPKM values. For downstream analyses and visualization, gene expression matrices were transformed as log2(FPKM + 1).

## S4.2 Differential gene expression analysis

Differential gene expression analysis between TSR-defined groups was performed using the DESeq2 R package. DESeq2 models count data using a negative binomial distribution and applies the Benjamini–Hochberg procedure to control the false discovery rate. Genes with adjusted p-values < 0.05 and |log2 fold change| ≥ 1 were considered significantly differentially expressed. Differentially expressed genes were visualized using volcano plots.

## S4.3 Functional enrichment analysis

To investigate the biological processes associated with TSR-related transcriptional alterations, functional enrichment analyses were conducted using the clusterProfiler R package. Gene Ontology (GO) enrichment analysis was performed to identify overrepresented biological processes, cellular components, and molecular functions. Kyoto Encyclopedia of Genes and Genomes (KEGG) pathway analysis was used to identify signaling pathways enriched in differentially expressed genes. Pathways with adjusted p-values < 0.05 were considered significantly enriched.

## S4.4 Gene set enrichment analysis

Gene Set Enrichment Analysis (GSEA) was conducted to evaluate pathway-level differences between TSR groups. Genes were ranked according to their differential expression statistics, and enrichment of predefined gene sets was evaluated using the GSEA algorithm. Gene sets from KEGG databases were analyzed independently, and pathways with significant enrichment scores and adjusted p-values were considered biologically relevant.

## S4.5 Gene signature scoring

To quantify pathway activity at the sample level, single-sample Gene Set Enrichment Analysis (ssGSEA) was performed. Hallmark gene sets were obtained from the Molecular Signatures Database (MSigDB). Enrichment scores were calculated for each sample to evaluate the activity of biological programs relevant to tumor microenvironment remodeling, including TGF-β signaling, epithelial–mesenchymal transition (EMT), and extracellular matrix (ECM) organization.

Associations between the radiomics-derived TSR score and pathway activity were assessed using correlation analysis.

## S4.6 Tumor microenvironment estimation

The composition of the tumor microenvironment was inferred from bulk transcriptomic data using several computational approaches. ESTIMATE was applied to calculate stromal scores, ESTIMATE scores, and tumor purity. In addition, immune cell infiltration was estimated using MCPcounter and quanTIseq, which quantify the abundance of multiple immune and stromal cell populations based on gene expression signatures.

# Appendix S5

## Preparation and analysis of single-cell RNA sequence

## S5.1 Preparation of Tissue

Freshly resected tumor tissues were immediately transferred into RNase-free PBS and washed to remove blood and debris. The tissues were minced into small fragments (~0.5 mm²) and enzymatically digested in a solution containing collagenase IV, papain, and DNase I. After digestion, the suspension was gently triturated and filtered through a cell strainer to obtain single-cell suspensions. Red blood cells and dead cells were removed, and viable cells were resuspended in PBS containing 0.04% BSA. Cell viability was assessed using trypan blue staining, and samples with viability >85% were used for downstream sequencing.

## S5.2 Single-cell library construction and sequencing workflow

Single-cell suspensions were processed using the 10x Genomics Chromium platform with the Single Cell 3’ Library Kit v3 according to the manufacturer’s protocol. Approximately 8,000 cells were targeted per channel. Libraries were sequenced on an Illumina NovaSeq 6000 platform with paired-end reads. Raw sequencing data were processed using CellRanger (v7.0.0) for alignment to the human reference genome and generation of gene–cell count matrices.

## S5.3 Data processing, clustering, and cell-type annotation

Single-cell RNA sequencing data were analyzed using the Seurat framework in R. Cells with fewer than 300 detected genes, fewer than 1,000 UMI counts, or mitochondrial transcript proportions greater than 20% were excluded during quality control. Potential doublets were further identified and removed using DoubletFinder.

After filtering, data from all samples were merged and normalized, and highly variable genes were identified for downstream analyses. Dimensionality reduction was performed using principal component analysis, followed by batch-effect correction with the Harmony algorithm. Cells were subsequently clustered using a graph-based approach and visualized using UMAP.

Cell type annotation was performed by combining automated prediction using SingleR with manual inspection of cluster-specific marker genes identified by the FindAllMarkers function. Major cell populations were first defined based on canonical marker genes, and further subclustering analyses were conducted for fibroblast and T-cell populations to characterize their transcriptional heterogeneity. Fibroblasts were further resolved into CAF states: LRRC15⁺ CAF (LRRC15, MMP11), iCAF (DPEP1, CST2), apCAF-like (CD74, HLA-DRA), quiescent CAF (SRGN, SDC1), stress-response CAF (HSPA1A, DNAJA1), ion-signaling CAF (GPC6, CACNA1C), immediate-early CAF (JUN, ATF3), and hCAF (GPBAR1, SFRP1). T cells were resolved into effector T cells (GZMB, NKG7), exhausted CD8⁺ T cells (TOX, CXCL13), progenitor-exhausted T cells (SLAMF6, THEMIS), regulatory T cells (IL2RA, TNFRSF4), central memory T cells (CCR7, KLF2), effector memory T cells (Tem; GZMK, CST7), and T follicular helper cells (Tfh). Representative subset markers are shown in Figure S6b and S6f.

# Supplementary table

## Table S1 MRI acquisition parameters across multi-centers

| **Center** | **MRI** | **Parameters** | **TR** | **TE** | **FOV** | **Matrix** | **Section thickness(mm)** | **Gap** |
| --- | --- | --- | --- | --- | --- | --- | --- | --- |
| **Center1** | 1.5T MR (MAGNETOM Aera, Siemens) | T2-weighted imaging | 4918 | 106 | 285×380 | 384×273 | 5.5 | 1.2 |
|  |  | DWI ^a^ | 5100 | 55 | 285×380 | 192×154 | 5.5 | 1.2 |
|  |  | T1-weighted in-phase and opposed-phase imaging | 6.88 | 2.39/4.77 | 356×380 | 320×240 | 3.5 | 0 |
|  |  | Contrast-enhanced  T1-weighted imaging | 3.47 | 1.36 | 308×380 | 320×240 | 3 | 0 |
|  | 3.0T MR (Prisma, Siemens) | T2-weighted imaging | 587 | 103 | 380×344 | 320×320 | 6 | 1.2 |
|  |  | DWI ^a^ | 5000 | 65 | 380×310 | 130×130 | 5 | 1.2 |
|  |  | T1-weighted in-phase and opposed-phase imaging | 3.97 | 1.29/2.52 | 380×285 | 320×288 | 3.0 | 0 |
|  |  | Contrast-enhanced  T1-weighted imaging | 3.10 | 1.23 | 380×309 | 320×288 | 3 | 0 |
| **Center 2** | Philips Ingenia CX 3.0T PHILIPS | T2-weighted imaging | 1250 | 70 | 36.2×36.2 | 212×216 | 5 | 0.5 |
|  |  | DWI^b^ | 3000 | 59.3 | 37.5×37.5 | 132×161 | 6.5 | 3.25 |
|  |  | T1-weighted in-phase and opposed-phase imaging | 165 | 2.3/1.15 | 34.8×34.8 | 152×194 | 7 | 0 |
|  |  | Contrast-enhanced  T1-weighted imaging | 3.03 | 1.42 | 34.5×34.5 | 232×264 | 4.6 | 0 |
| **Center 3** | 1.5T MR (Amira, Siemens) | T2-weighted imaging | 2000 | 85 | 390×390 | 325×325 | 5.5 | 1.1 |
|  |  | DWI ^a^ | 4200 | 55 | 400×275 | 267×183 | 5.5 | 1.1 |
|  |  | T1-weighted in-phase and opposed-phase imaging | 140 | 2.38/4.77 | 380×285 | 633×475 | 5.5 | 1.1 |
|  |  | Contrast-enhanced  T1-weighted imaging | 3.92 | 1.46 | 390×305 | 325×254 | 3 | 0.6 |
|  | 3.0T MR (Ingenia, Philips) | T2-weighted imaging | 8365 | 90 | 390×390 | 300×300 | 5 | 1 |
|  |  | DWI ^a^ | 3368 | 67.5 | 360×310 | 120×90 | 5 | 1 |
|  |  | T1-weighted in-phase and opposed-phase imaging | 180 | 1.15/2.3 | 400×310 | 224×171 | 5 | 1 |
|  |  | Contrast-enhanced  T1-weighted imaging | 3.1 | 1.11 | 390×340 | 228×200 | 5 | -2.5 |

Note. — TR = Repetition time; TE = Echo time; FOV = Field of view;

DWI = Diffusion-weighted Imaging;

^a^ performed with b values of 0 and 500 sec/mm^2^

^b^ performed with b values of 0, 800 sec/mm^2^

## Table S2 Patient demographics and clinicopathological variables across TSR subgroups

| **Parameters** | **TT (n=166)** |  |  |  | **ETV (n=92)** |  |  |
| --- | --- | --- | --- | --- | --- | --- | --- |
|  | L-TSR (n=51) | H-TSR (n=108) | *P* |  | L-TSR (n=34) | H-TSR (n=58) | *P* |
| **Age (years)** | 66.72 ± 9.81 | 64.16 ± 8.02 | 0.071 |  | 63.50 [56.00, 74.00] | 66.00 [58.00, 70.00] | 0.814 |
| **Sex** |  |  | 0.820 |  |  |  | 0.847 |
| **Female** | 26 (44.83%) | 45 (41.67%) |  |  | 12 (35.29%) | 23 (39.66%) |  |
| **Male** | 32 (55.17%) | 63 (58.33%) |  |  | 22 (64.71%) | 35 (60.34%) |  |
| **DM** |  |  | 0.360 |  |  |  | >0.999 |
| **Absence** | 36 (62.07%) | 76 (70.37%) |  |  | 23 (67.65%) | 38 (65.52%) |  |
| **Presence** | 22 (37.93%) | 32 (29.63%) |  |  | 11 (32.35%) | 20 (34.48%) |  |
| **HBP** |  |  | 0.923 |  |  |  | 0.557 |
| **Absence** | 31 (53.45%) | 60 (55.56%) |  |  | 20 (58.82%) | 39 (67.24%) |  |
| **Presence** | 27 (46.55%) | 48 (44.44%) |  |  | 14 (41.18%) | 19 (32.76%) |  |
| **BMI (kg/m²)** | 22.07 ± 3.13 | 22.62 ± 3.16 | 0.285 |  | 23.02 ± 2.66 | 22.92 ± 3.49 | 0.891 |
| **TB (μmol/L)** |  |  | 0.804 |  |  |  | 0.371 |
| **≤ 20.4** | 40 (68.97%) | 71 (65.74%) |  |  | 25 (73.53%) | 36 (62.07%) |  |
| **> 20.4** | 18 (31.03%) | 37 (34.26%) |  |  | 9 (26.47%) | 22 (37.93%) |  |
| **Albumin (g/L)** |  |  | >0.999 |  |  |  | 0.264 |
| **> 35** | 53 (91.38%) | 100 (92.59%) |  |  | 33 (97.06%) | 51 (87.93%) |  |
| **≤ 35** | 5 (8.62%) | 8 (7.41%) |  |  | 1 (2.94%) | 7 (12.07%) |  |
| **CA19-9 (U/mL)** |  |  | 0.290 |  |  |  | 0.078 |
| **≤ 100** | 31 (53.45%) | 47 (43.52%) |  |  | 17 (50.00%) | 17 (29.31%) |  |
| **> 100** | 27 (46.55%) | 61 (56.48%) |  |  | 17 (50.00%) | 41 (70.69%) |  |
| **Location** |  |  | 0.441 |  |  |  | >0.999 |
| **Head/uncinate** | 30 (51.72%) | 64 (59.26%) |  |  | 20 (58.82%) | 33 (56.90%) |  |
| **Body/tail** | 28 (48.28%) | 44 (40.74%) |  |  | 14 (41.18%) | 25 (43.10%) |  |
| **Tumor size (cm)** | 2.30 [2.00, 3.00] | 2.80 [2.10, 3.40] | 0.044 |  | 2.50 [1.80, 3.10] | 3.25 [2.40, 3.80] | 0.007 |
| **pT Stage** |  |  | 0.156 |  |  |  | 0.655 |
| **T1** | 21 (36.21%) | 27 (25.00%) |  |  | 8 (23.53%) | 12 (20.69%) |  |
| **T2** | 27 (46.55%) | 67 (62.04%) |  |  | 19 (55.88%) | 29 (50.00%) |  |
| **T3** | 10 (17.24%) | 14 (12.96%) |  |  | 7 (20.59%) | 17 (29.31%) |  |
| **pN Stage** |  |  | 0.610 |  |  |  | 0.759 |
| **N0** | 31 (53.45%) | 58 (53.70%) |  |  | 21 (61.76%) | 38 (65.52%) |  |
| **N1** | 24 (41.38%) | 40 (37.04%) |  |  | 7 (20.59%) | 13 (22.41%) |  |
| **N2** | 3 (5.17%) | 10 (9.26%) |  |  | 6 (17.65%) | 7 (12.07%) |  |
| **PD** |  |  | 0.708 |  |  |  | 0.446 |
| **Well/moderately-differentiated** | 31 (53.45%) | 53 (49.07%) |  |  | 20 (58.82%) | 28 (48.28%) |  |
| **Poor** | 27 (46.55%) | 55 (50.93%) |  |  | 14 (41.18%) | 30 (51.72%) |  |
| **PFI** |  |  | 0.716 |  |  |  | 0.054 |
| **Absence** | 14 (24.14%) | 22 (20.37%) |  |  | 12 (35.29%) | 9 (15.52%) |  |
| **Presence** | 44 (75.86%) | 86 (79.63%) |  |  | 22 (64.71%) | 49 (84.48%) |  |
| **PVI** |  |  | 0.773 |  |  |  | 0.098 |
| **Absence** | 37 (63.79%) | 65 (60.19%) |  |  | 27 (79.41%) | 35 (60.34%) |  |
| **Presence** | 21 (36.21%) | 43 (39.81%) |  |  | 7 (20.59%) | 23 (39.66%) |  |
| **PNI** |  |  | 0.716 |  |  |  | 0.084 |
| **Absence** | 14 (24.14%) | 22 (20.37%) |  |  | 9 (26.47%) | 6 (10.34%) |  |
| **Presence** | 44 (75.86%) | 86 (79.63%) |  |  | 25 (73.53%) | 52 (89.66%) |  |
| **AC** |  |  | 0.126 |  |  |  | 0.685 |
| **Without AC** | 13 (22.41%) | 13 (12.04%) |  |  | 4 (11.76%) | 10 (17.24%) |  |
| **With AC** | 45 (77.59%) | 95 (87.96%) |  |  | 30 (88.24%) | 48 (82.76%) |  |
| **RFS (months)** | 21.15 [8.00, 42.30] | 13.20 [6.80, 28.40] | 0.065 |  | 7.42 [3.87, 12.87] | 5.12 [3.30, 9.87] | 0.165 |
| **Recurrence** |  |  | 0.116 |  |  |  | <0.001 |
| **No** | 25 (43.10%) | 32 (29.63%) |  |  | 23 (67.65%) | 17 (29.31%) |  |
| **Yes** | 33 (56.90%) | 76 (70.37%) |  |  | 11 (32.35%) | 41 (70.69%) |  |
| **TSR Radscore** | 0.48 [0.28, 0.68] | 0.85 [0.74, 0.91] | <0.001 |  | 0.59 [0.30, 0.77] | 0.79 [0.68, 0.90] | <0.001 |

TT: TSR training, ETV: external TSR validation; PDAC = pancreatic ductal adenocarcinoma, DM= diabetes, HBP= high blood pressure, BMI = body mass index, TB = total bilirubin, CA19-9= cancer antigen 19-9, PD = pathological Differentiation, PVI=pathological lymphovascular invasion; PFI= peripancreatic fat invasion, PNI= perineural invasion. AC= Adjuvant treatment. RFS= recurrence-free survival

## Table S3 Characteristics in LAPC validation cohort stratified by srMRI-based model

| **Variables** |  | **Overall (n=82)** | **H-TSR (n=35)** | **L-TSR (n=47)** | **P value** |
| --- | --- | --- | --- | --- | --- |
| **Age (years) ^b^** |  | 68.00 [59.00, 75.00] | 65.00 [57.00, 75.00] | 70.00 [65.00, 75.50] | 0.104 |
| **Location ^a^** | Body/tail | 17 (20.7%) | 9 (25.7%) | 8 (17.0%) | 0.493 |
|  | Head/uncinate | 65 (79.3%) | 26 (74.3%) | 39 (83.0%) |  |
| **Albumin ^a^** | ≥35μmol L^−1^ | 78 (95.1%) | 35 (100.0%) | 43 (91.5%) | 0.211 |
|  | <35μmol L^−1^ | 4 (4.9%) | 0 (0.0%) | 4 (8.5%) |  |
| **Sex ^a^** | Female | 38 (46.3%) | 18 (42.9%) | 20 (50.0%) | 0.669 |
|  | Male | 44 (53.7%) | 24 (57.1%) | 20 (50.0%) |  |
| **CA199 ^a^** | >100 U ml^−1^ | 46 (56.1%) | 20 (57.1%) | 26 (55.3%) | 0.898 |
|  | ≤100 U ml^−1^ | 36 (43.9%) | 15 (42.9%) | 21 (44.7%) |  |
| **TB ^a^** | >22.2μmol L^−1^ | 21 (25.6%) | 5 (14.3%) | 16 (34.0%) | 0.076 |
|  | ≤22.2μmol L^−1^ | 61 (74.4%) | 30 (85.7%) | 31 (66.0%) |  |
| **HBP ^a^** | Absence | 43 (52.4%) | 22 (62.9%) | 21 (44.7%) | 0.160 |
|  | Presence | 39 (47.6%) | 13 (37.1%) | 26 (55.3%) |  |
| **DM ^a^** | Absence | 58 (70.7%) | 24 (68.6%) | 34 (72.3%) | 0.900 |
|  | Presence | 24 (29.3%) | 11 (31.4%) | 13 (27.7%) |  |
| **Tumor size (cm) ^b^** |  | 3.20 [2.70, 3.80] | 3.20 [2.95, 4.10] | 3.20 [2.40, 3.60] | 0.219 |
| **T stage ^a^** |  |  |  |  |  |
|  | T2 | 14 (17.1%) | 8 (22.9%) | 6 (12.8%) | 0.463 |
|  | T3 | 9 (11.0%) | 4 (11.4%) | 5 (10.6%) |  |
|  | T4 | 59 (72.0%) | 23 (65.7%) | 36 (76.6%) |  |
| **Immunotherapy ^a^** | No | 45 (54.9%) | 17 (48.6%) | 28 (59.6%) | 0.444 |
|  | Yes | 37 (45.1%) | 18 (51.4%) | 19 (40.4%) |  |
| **TSR radscore** |  | 0.55 [0.43, 0.83] | 0.84 [0.79, 0.92] | 0.44 [0.34, 0.53] | **<0.001** |
| **PFS time (months) ^b^** |  | 7.63 [5.05, 11.30] | 8.00 [5.40, 11.75] | 7.10 [4.57, 11.05] | 0.391 |
| **PFS event ^a^** | No progression | 27 (32.9%) | 17 (48.6%) | 10 (21.3%) | **0.018** |
|  | Progression | 55 (67.1%) | 18 (51.4%) | 37 (78.7%) |  |
| **OS time (months) ^b^** |  | 14.80 [10.68, 19.08] | 14.61 [10.08, 18.57] | 15.90 [10.70, 19.25] | 0.600 |
| **OS event ^a^** | Alive | 45 (54.9%) | 26 (74.3%) | 19 (40.4%) | **0.005** |
|  | Dead | 37 (45.1%) | 9 (25.7%) | 28 (59.6%) |  |

^a^ median (interquartile ranges), ^b^ Number of patients (percentage)

LAPC = locally advanced pancreatic cancer, CA 19-9= cancer antigen 19-9, TB = total bilirubin, BMI = body mass index, DM = diabetes, HBP = high blood pressure

## Table S4 Performance of srMRI Radiomics Models from Single Sequence

|  | **TT cohort** | | |  |  |  | **ETV cohort** | | |  |  |  |
| --- | --- | --- | --- | --- | --- | --- | --- | --- | --- | --- | --- | --- |
| **Models** | **Accuracy** | **AUC(95% CI)** | **Sensitivity** | **Specificity** | **Precision** | **F1 Score** | **Accuracy** | **AUC(95% CI)** | **Sensitivity** | **Specificity** | **Precision** | **F1 Score** |
| **RDM_DWI_ExtraTrees** | 0.633 | 0.696(0.616 - 0.776) | 0.546 | 0.793 | 0.831 | 0.659 | 0.609 | 0.602(0.484 - 0.720) | 0.655 | 0.529 | 0.704 | 0.679 |
| **RDM_DWI_KNN** | 0.608 | 0.689(0.608 - 0.770) | 0.537 | 0.741 | 0.795 | 0.641 | 0.587 | 0.645(0.531 - 0.758) | 0.448 | 0.824 | 0.813 | 0.578 |
| **RDM_DWI_LR** | 0.681 | 0.653(0.569 - 0.737) | 0.815 | 0.431 | 0.727 | 0.769 | 0.554 | 0.566(0.446 - 0.686) | 0.776 | 0.176 | 0.616 | 0.687 |
| **RDM_DWI_NaiveBayes** | 0.645 | 0.689(0.609 - 0.770) | 0.620 | 0.690 | 0.788 | 0.694 | 0.609 | 0.645(0.531 - 0.758) | 0.690 | 0.471 | 0.690 | 0.690 |
| **RDM_DWI_RandomForest** | 0.711 | 0.644(0.559 - 0.729) | 0.861 | 0.431 | 0.738 | 0.795 | 0.630 | 0.574(0.454 - 0.694) | 0.862 | 0.235 | 0.658 | 0.746 |
| **RDM_DWI_SVM** | 0.633 | 0.644(0.559 - 0.729) | 0.657 | 0.586 | 0.747 | 0.700 | 0.565 | 0.583(0.464 - 0.702) | 0.741 | 0.265 | 0.632 | 0.683 |
| **RDM_DWI_XGBoost** | 0.681 | 0.688(0.608 - 0.769) | 0.676 | 0.690 | 0.802 | 0.734 | 0.609 | 0.609(0.492 - 0.726) | 0.741 | 0.382 | 0.672 | 0.705 |
| **RDM_T2_ExtraTrees** | 0.602 | 0.650(0.566 - 0.735) | 0.574 | 0.655 | 0.756 | 0.653 | 0.630 | 0.726(0.623 - 0.828) | 0.603 | 0.676 | 0.761 | 0.673 |
| **RDM_T2_KNN** | 0.645 | 0.699(0.620 - 0.779) | 0.537 | 0.845 | 0.866 | 0.663 | 0.554 | 0.652(0.539 - 0.764) | 0.414 | 0.794 | 0.774 | 0.539 |
| **RDM_T2_LR** | 0.596 | 0.699(0.620 - 0.779) | 0.472 | 0.828 | 0.836 | 0.604 | 0.522 | 0.677(0.568 - 0.787) | 0.414 | 0.706 | 0.706 | 0.522 |
| **RDM_T2_NaiveBayes** | 0.560 | 0.679(0.597 - 0.761) | 0.380 | 0.897 | 0.872 | 0.529 | 0.293 | 0.537(0.415 - 0.658) | 0.086 | 0.647 | 0.294 | 0.133 |
| **RDM_T2_RandomForest** | 0.627 | 0.666(0.582 - 0.749) | 0.556 | 0.759 | 0.811 | 0.659 | 0.522 | 0.599(0.482 - 0.717) | 0.414 | 0.706 | 0.706 | 0.522 |
| **RDM_T2_SVM** | 0.639 | 0.691(0.610 - 0.771) | 0.602 | 0.707 | 0.793 | 0.684 | 0.630 | 0.718(0.614 - 0.822) | 0.672 | 0.559 | 0.722 | 0.696 |
| **RDM_T2_XGBoost** | 0.729 | 0.666(0.582 - 0.749) | 0.880 | 0.448 | 0.748 | 0.809 | 0.620 | 0.602(0.485 - 0.720) | 0.810 | 0.294 | 0.662 | 0.729 |
| **RDM_T1PRE_ExtraTrees** | 0.675 | 0.633(0.547 - 0.719) | 0.852 | 0.345 | 0.708 | 0.773 | 0.587 | 0.620(0.504 - 0.736) | 0.793 | 0.235 | 0.639 | 0.708 |
| **RDM_T1PRE_KNN** | 0.614 | 0.671(0.588 - 0.753) | 0.583 | 0.672 | 0.768 | 0.663 | 0.576 | 0.612(0.495 - 0.729) | 0.500 | 0.706 | 0.744 | 0.598 |
| **RDM_T1PRE_LR** | 0.663 | 0.618(0.531 - 0.705) | 0.759 | 0.483 | 0.732 | 0.745 | 0.576 | 0.544(0.423 - 0.665) | 0.690 | 0.382 | 0.656 | 0.672 |
| **RDM_T1PRE_NaiveBayes** | 0.675 | 0.598(0.510 - 0.686) | 0.815 | 0.414 | 0.721 | 0.765 | 0.609 | 0.578(0.459 - 0.697) | 0.741 | 0.382 | 0.672 | 0.705 |
| **RDM_T1PRE_RandomForest** | 0.639 | 0.655(0.571 - 0.739) | 0.667 | 0.586 | 0.750 | 0.706 | 0.598 | 0.611(0.494 - 0.727) | 0.621 | 0.559 | 0.706 | 0.661 |
| **RDM_T1PRE_SVM** | 0.488 | 0.420(0.328 - 0.512) | 0.565 | 0.345 | 0.616 | 0.589 | 0.533 | 0.498(0.375 - 0.621) | 0.638 | 0.353 | 0.627 | 0.632 |
| **RDM_T1PRE_XGBoost** | 0.578 | 0.689(0.609 - 0.770) | 0.407 | 0.897 | 0.880 | 0.557 | 0.511 | 0.646(0.533 - 0.759) | 0.310 | 0.853 | 0.783 | 0.444 |
| **RDM_T1A_ExtraTrees** | 0.602 | 0.695(0.615 - 0.775) | 0.500 | 0.793 | 0.818 | 0.621 | 0.424 | 0.513(0.391 - 0.636) | 0.276 | 0.676 | 0.593 | 0.376 |
| **RDM_T1A_KNN** | 0.669 | 0.642(0.557 - 0.727) | 0.769 | 0.483 | 0.735 | 0.751 | 0.576 | 0.630(0.515 - 0.745) | 0.724 | 0.324 | 0.646 | 0.683 |
| **RDM_T1A_LR** | 0.566 | 0.628(0.542 - 0.715) | 0.472 | 0.741 | 0.773 | 0.586 | 0.435 | 0.542(0.421 - 0.664) | 0.310 | 0.647 | 0.600 | 0.409 |
| **RDM_T1A_NaiveBayes** | 0.645 | 0.655(0.570 - 0.739) | 0.676 | 0.586 | 0.753 | 0.712 | 0.565 | 0.609(0.492 - 0.726) | 0.552 | 0.588 | 0.696 | 0.615 |
| **RDM_T1A_RandomForest** | 0.620 | 0.663(0.579 - 0.746) | 0.583 | 0.690 | 0.778 | 0.667 | 0.533 | 0.608(0.491 - 0.725) | 0.500 | 0.588 | 0.674 | 0.574 |
| **RDM_T1A_SVM** | 0.645 | 0.391(0.300 - 0.483) | 0.981 | 0.017 | 0.650 | 0.782 | 0.620 | 0.406(0.284 - 0.528) | 0.983 | 0.000 | 0.626 | 0.765 |
| **RDM_T1A_XGBoost** | 0.717 | 0.662(0.579 - 0.745) | 0.815 | 0.534 | 0.765 | 0.789 | 0.609 | 0.599(0.482 - 0.717) | 0.724 | 0.412 | 0.677 | 0.700 |
| **RDM_T1V_ExtraTrees** | 0.687 | 0.711(0.633 - 0.790) | 0.741 | 0.586 | 0.769 | 0.755 | 0.511 | 0.529(0.407 - 0.651) | 0.603 | 0.353 | 0.614 | 0.609 |
| **RDM_T1V_KNN** | 0.620 | 0.685(0.604 - 0.766) | 0.509 | 0.828 | 0.846 | 0.636 | 0.565 | 0.616(0.500 - 0.732) | 0.466 | 0.735 | 0.750 | 0.574 |
| **RDM_T1V_LR** | 0.681 | 0.697(0.617 - 0.777) | 0.769 | 0.517 | 0.748 | 0.758 | 0.587 | 0.581(0.461 - 0.700) | 0.638 | 0.500 | 0.685 | 0.661 |
| **RDM_T1V_NaiveBayes** | 0.687 | 0.710(0.632 - 0.789) | 0.741 | 0.586 | 0.769 | 0.755 | 0.609 | 0.591(0.473 - 0.710) | 0.638 | 0.559 | 0.712 | 0.673 |
| **RDM_T1V_RandomForest** | 0.687 | 0.738(0.663 - 0.813) | 0.630 | 0.793 | 0.850 | 0.723 | 0.565 | 0.652(0.539 - 0.764) | 0.483 | 0.706 | 0.737 | 0.583 |
| **RDM_T1V_SVM** | 0.699 | 0.660(0.576 - 0.743) | 0.889 | 0.345 | 0.716 | 0.793 | 0.641 | 0.577(0.457 - 0.696) | 0.810 | 0.353 | 0.681 | 0.740 |
| **RDM_T1V_XGBoost** | 0.651 | 0.689(0.609 - 0.770) | 0.593 | 0.759 | 0.821 | 0.688 | 0.554 | 0.630(0.515 - 0.745) | 0.552 | 0.559 | 0.681 | 0.610 |
| **RDM_T1D_ExtraTrees** | 0.620 | 0.657(0.573 - 0.741) | 0.574 | 0.707 | 0.785 | 0.663 | 0.543 | 0.557(0.436 - 0.678) | 0.483 | 0.647 | 0.700 | 0.571 |
| **RDM_T1D_KNN** | 0.711 | 0.663(0.580 - 0.746) | 0.870 | 0.414 | 0.734 | 0.797 | 0.609 | 0.614(0.497 - 0.730) | 0.845 | 0.206 | 0.645 | 0.731 |
| **RDM_T1D_LR** | 0.723 | 0.683(0.602 - 0.764) | 0.917 | 0.362 | 0.728 | 0.811 | 0.652 | 0.653(0.540 - 0.765) | 0.862 | 0.294 | 0.676 | 0.758 |
| **RDM_T1D_NaiveBayes** | 0.620 | 0.689(0.608 - 0.770) | 0.593 | 0.672 | 0.771 | 0.670 | 0.609 | 0.641(0.528 - 0.755) | 0.621 | 0.588 | 0.720 | 0.667 |
| **RDM_T1D_RandomForest** | 0.651 | 0.697(0.617 - 0.777) | 0.602 | 0.741 | 0.813 | 0.691 | 0.674 | 0.663(0.551 - 0.774) | 0.621 | 0.765 | 0.818 | 0.706 |
| **RDM_T1D_SVM** | 0.663 | 0.662(0.579 - 0.746) | 0.759 | 0.483 | 0.732 | 0.745 | 0.641 | 0.675(0.566 - 0.785) | 0.759 | 0.441 | 0.698 | 0.727 |
| **RDM_T1D_XGBoost** | 0.675 | 0.673(0.591 - 0.756) | 0.778 | 0.483 | 0.737 | 0.757 | 0.630 | 0.608(0.490 - 0.725) | 0.672 | 0.559 | 0.722 | 0.696 |

## Table S5 Performance of srMRI Model Using Different Machine Learning Algorithms for Predicting Tumor-Stroma Ratio

| **Cohorts** | **Model** | **ACC** | **AUC (95%CI)** | **SEN** | **SPEC** | **PPV** | **NPV** | **F1-score** |
| --- | --- | --- | --- | --- | --- | --- | --- | --- |
| **TT** |  |  |  |  |  |  |  |  |
|  | ExtraTrees | 0.723 | 0.803(0.737 - 0.868) | 0.713 | 0.741 | 0.837 | 0.581 | 0.770 |
|  | KNN | 0.807 | 0.839(0.781 - 0.898) | 0.880 | 0.672 | 0.833 | 0.750 | 0.856 |
|  | LR | 0.807 | 0.833(0.773 - 0.893) | 0.824 | 0.776 | 0.873 | 0.703 | 0.848 |
|  | NaiveBayes | 0.795 | 0.821(0.759 - 0.884) | 0.806 | 0.776 | 0.870 | 0.682 | 0.837 |
|  | RandomForest | 0.819 | 0.837(0.778 - 0.896) | 0.852 | 0.759 | 0.868 | 0.733 | 0.860 |
|  | SVM | 0.825 | 0.837(0.778 - 0.897) | 0.889 | 0.707 | 0.850 | 0.774 | 0.869 |
|  | XGBoost | 0.849 | 0.870(0.817 - 0.922) | 0.917 | 0.724 | 0.861 | 0.824 | 0.888 |
| **ETV** |  |  |  |  |  |  |  |  |
|  | ExtraTrees | 0.620 | 0.652(0.539 - 0.764) | 0.603 | 0.647 | 0.745 | 0.489 | 0.667 |
|  | KNN | 0.685 | 0.740(0.640 - 0.840) | 0.793 | 0.500 | 0.730 | 0.586 | 0.760 |
|  | LR | 0.717 | 0.710(0.605 - 0.815) | 0.793 | 0.588 | 0.767 | 0.625 | 0.780 |
|  | NaiveBayes | 0.620 | 0.758(0.661 - 0.855) | 0.724 | 0.441 | 0.689 | 0.484 | 0.706 |
|  | RandomForest | 0.685 | 0.761(0.664 - 0.857) | 0.707 | 0.647 | 0.774 | 0.564 | 0.739 |
|  | SVM | 0.728 | 0.769(0.674 - 0.864) | 0.862 | 0.500 | 0.746 | 0.680 | 0.800 |
|  | XGBoost | 0.739 | 0.791(0.700 - 0.881) | 0.828 | 0.588 | 0.774 | 0.667 | 0.800 |

## Table S6 Uni- and Multivariate Regression of Independent Risk Factors for Recurrence

|  | **Univariate Analysis** |  |  | **Multivariate Analysis** |  |
| --- | --- | --- | --- | --- | --- |
| Parameters | HR (95% CI) | *P* value |  | HR (95% CI) | *P* value |
| Age (years) | 1.00 (0.98-1.02) | 0.835 |  |  |  |
| Sex [Male] | 1.31 (0.89-1.93) | 0.167 |  |  |  |
| DM [Presence] | 1.13 (0.76-1.68) | 0.530 |  |  |  |
| HBP [Presence] | 0.89 (0.59-1.35) | 0.590 |  |  |  |
| BMI (kg/m²) | 1.23 (0.84-1.79) | 0.283 |  |  |  |
| TB (>20.4 μmol/L) | 0.97 (0.91-1.03) | 0.284 |  |  |  |
| Albumin (≤35 g/L) | 0.89 (0.41-1.91) | 0.759 |  |  |  |
| CA19-9 (>100 U/mL) | 1.75 (1.19-2.57) | **0.004** |  | 1.25 (0.83–1.88) | 0.283 |
| Location [Body/tail] | 0.82 (0.56-1.20) | 0.313 |  |  |  |
| Tumor size (cm) | 1.40 (1.21-1.62) | **<0.001** |  | 1.38 (1.18–1.61) | **<0.001** |
| pT Stage [T2/3] | 1.09 (0.72-1.65) | 0.688 |  |  |  |
| pN Stage [N1/2] | 1.68 (1.15-2.45) | **0.007** |  | 1.52 (1.03–2.23) | **0.033** |
| PD [Poor] | 1.53 (1.05-2.24) | **0.027** |  | 1.35 (0.91–2.00) | 0.139 |
| PFI [Presence] | 1.79 (1.10-2.90) | **0.018** |  | 1.72 (1.06–2.80) | **0.028** |
| PVI [Presence] | 1.55 (1.06-2.27) | **0.023** |  | 1.24 (0.80–1.91) | 0.338 |
| PNI [Presence] | 1.14 (0.66-1.97) | 0.641 |  |  |  |
| AC [yes] | 0.78 (0.44-1.40) | 0.407 |  |  |  |
| TSR radscore | 3.93 (1.59-9.68) | **0.003** |  | 3.12 (1.29–7.51) | **0.011** |

Bold values indicate statistical significance.

PDAC = pancreatic ductal adenocarcinoma, DM= diabetes, HBP= high blood pressure, BMI = body mass index, TB = total bilirubin, CA19-9= cancer antigen 19-9, PD = pathological Differentiation, PVI=pathological lymphovascular invasion; PFI= peripancreatic fat invasion, PNI= perineural invasion. AC= Adjuvant treatment. RFS= recurrence-free survival

## Table S7. Treatment distribution and survival outcomes by chemotherapy backbone in the LAPC cohort

| **Treatment group** | **Chemotherapy backbone** | **n** | **Median PFS, months [IQR]** | **Median OS, months [IQR]** |
| --- | --- | --- | --- | --- |
| Chemotherapy alone | AG | 24 | 7.4 [5.0, 10.8] | 13.6 [8.0, 17.9] |
|  | AS | 5 | 6.5 [4.2, 6.7] | 13.9 [10.7, 14.6] |
|  | GS | 7 | 3.1 [2.7, 5.3] | 11.8 [10.8, 16.7] |
|  | Gemcitabine | 1 | 7.8 [NA, NA] | 19.5 [NA, NA] |
|  | S-1 | 8 | 8.6 [6.8, 10.8] | 13.0 [9.8, 17.1] |
| Chemotherapy + anti-PD-1 | AG | 28 | 9.6 [6.4, 15.8] | 17.4 [12.7, 24.1] |
|  | AS | 3 | 6.0 [5.5, 8.1] | 14.4 [9.7, 18.9] |
|  | Gemcitabine | 2 | 6.3 [5.9, 6.7] | 11.0 [10.5, 11.5] |
|  | S-1 | 4 | 10.2 [8.2, 11.3] | 11.7 [11.5, 13.1] |

Values are median [interquartile range] of PFS/OS time. PFS, progression-free survival; OS, overall survival; IQR, interquartile range. AG, albumin-bound paclitaxel plus gemcitabine; AS, albumin-bound paclitaxel plus S-1; GS, gemcitabine plus S-1. The interquartile range was not estimable for single-patient subgroups and is reported as NA.

## Table S8 Baseline characteristics of the LAPC cohort by ICI treatment and covariate balance before and after IPTW

| **Variables** |  | **non-ICI (n=45)** | **ICI (n=37)** | **P value** | **SMD*** |
| --- | --- | --- | --- | --- | --- |
| Age (years) ᵇ |  | 69.22 (9.46) | 65.41 (9.52) | 0.074 | 0.407 / 0.032 |
| Sex ᵃ | Female | 22 (48.9%) | 16 (43.2%) | 0.774 | 0.113 / 0.012 |
|  | Male | 23 (51.1%) | 21 (56.8%) |  |  |
| CA19-9 ᵃ | ≤100 U ml⁻¹ | 20 (44.4%) | 16 (43.2%) | 1.000 | 0.024 / 0.009 |
|  | >100 U ml⁻¹ | 25 (55.6%) | 21 (56.8%) |  |  |
| Tumor size (cm) ᵇ |  | 3.31 (1.20) | 3.57 (1.33) | 0.367 | 0.205 / 0.000 |
| Location ᵃ | Head/uncinate | 34 (75.6%) | 31 (83.8%) | 0.522 | 0.206 / 0.041 |
|  | Body/tail | 11 (24.4%) | 6 (16.2%) |  |  |

Data are presented as mean (SD) for continuous variables and n (%) for categorical variables.

* SMD values are shown as before / after IPTW weighting; balance was assessed by the absolute value, with |SMD| < 0.1 indicating adequate balance.

ᵃ n (%); ᵇ mean (SD).

LAPC = locally advanced pancreatic cancer; ICI = immune checkpoint inhibitor; IPTW = inverse-probability-of-treatment weighting; SMD = standardized mean difference; CA19-9 = carbohydrate antigen 19-9.

The IPTW-weighted Cox regression analysis yielded similar results, with weighted HRs of 0.47 (95% CI, 0.23–0.96; P = 0.039) for OS and 0.59 (95% CI, 0.33–1.04; P = 0.068) for PFS. Formal TSR-by-treatment interaction testing yielded P values of 0.179 for OS and 0.096 for PFS.

# Supplementary Figures

**Figure S1. Comparison of original and super-resolution (SR) MRI images across six sequences in a 74-year-old female patient with a low tumor–stroma ratio (TSR ≤1, TSR radscore=0.408).** For each MRI sequence, the native image (left) is displayed alongside its SR counterpart (right), with red markers outlining the region of interest and magnified crops shown beneath. The SR outputs provide visibly finer anatomical detail and higher spatial clarity while maintaining strong correspondence with the original images, as reflected by the accompanying peak signal-to-noise ratio (PSNR) and structural similarity index (SSIM) metrics.

**Figure S2. Comparison of original and super-resolution (SR) MRI images across six sequences in a 79-year-old female patient with a high tumor-stroma ratio (TSR > 1, TSR radscore=0.855).** For each MRI sequence, the native image (left) is displayed alongside its SR counterpart (right), with red markers outlining the region of interest and magnified crops shown beneath. The SR outputs provide visibly finer anatomical detail and higher spatial clarity while maintaining strong correspondence with the original images, as reflected by the accompanying peak signal-to-noise ratio (PSNR) and structural similarity index (SSIM) metrics.

**Figure S3. Model interpretability and TSR prediction ability of the srMRI-based TSR-radscore.** (A) SHAP summary plot illustrating the top contributing intensity- and texture-based radiomics features selected for the final srMRI model in the TSR-training (TT) and external TSR validation (ETV) cohorts. Positive SHAP values indicate stronger contributions toward the stroma-poor phenotype (TSR > 1), while negative values indicate alignment with the stroma-rich category. (B) Calibration curves in the TT ETV cohorts demonstrating good agreement between predicted and observed probabilities. (C) Decision curve analysis (DCA) showing that the srMRI model provides higher net clinical benefit across a range of clinically relevant threshold probabilities compared with treat-all and treat-none strategies in both cohorts.

**Figure S4.** Waterfall plots showing the best percentage change in target lesions among patients receiving (A) chemotherapy plus anti-PD-1 therapy and (B) chemotherapy alone..

**Figure S5. Transcriptomic characteristics associated with srMRI-derived TSR phenotypes.** (A) ssGSEA scores of stromal-related signatures (ECM structural signature, Hallmark EMT, and TGF-β signaling) were significantly higher in L-TSR tumors than in H-TSR tumors. (B) Immune deconvolution analysis showed increased fibroblast infiltration and cytotoxic lymphocyte signals in L-TSR tumors, whereas H-TSR tumors exhibited relatively lower stromal infiltration (red: H-TSR; blue: L-TSR; Wilcoxon test, *P < 0.05, **P < 0.01, ***P < 0.001).

**Figure S6 srMRI model-derived TSR-associated heterogeneity of CAF and T-cell subsets in PDAC.** (A) UMAP visualization of CAF subclusters identified from single-cell RNA sequencing, including LRRC15⁺ CAF, ion-signaling CAF, quiescent CAF, apCAF-like, stress-response CAF, hCAF, iCAF, and immediate-early CAF. (B) Heatmap showing representative marker genes across CAF subclusters. (C) Stacked bar plots illustrating the proportional distribution of CAF subtypes between L-TSR and H-TSR tumors and across individual samples. (D) Boxplots showing differences in representative CAF subsets between TSR phenotypes. (E) UMAP visualization of T-cell subpopulations, including Tex, effector T, progenitor exhausted T, Treg, central memory T, Tem, and Tfh cells. (F) Heatmap of representative marker genes defining each T-cell subset. (G) Stacked bar plots comparing T-cell subset composition between L-TSR and H-TSR groups and across samples. (H) Boxplots highlighting representative T-cell subsets enriched in different TSR phenotypes.
